# Synthesis, anticancer evaluation of novel hybrid pyrazole-based chalcones, molecular docking, DNA fragmentation, and gene expression: *in vitro* studies†

**DOI:** 10.1039/d4ra03375b

**Published:** 2024-07-09

**Authors:** Norhan Yasser, Farid M. Sroor, Haidan M. El-Shorbagy, Shaymaa M. Eissa, Hamdi M. Hassaneen, Ismail A. Abdelhamid

**Affiliations:** a Department of Zoology, Faculty of Science, Cairo University 12613 Giza Egypt; b Organometallic and Organometalloid Chemistry Department, National Research Centre 12622 Cairo Egypt faridsroor@gmx.de fm.sroor@nrc.sci.eg; c Department of Chemistry, Faculty of Science, Cairo University Giza Egypt ismail_shafy@yahoo.com ismail_shafy@cu.edu.eg; d Faculty of Biotechnology, October University for Modern Science and Arts 6th October Giza Egypt

## Abstract

A unique series of pyrazolyl-chalcone derivatives was synthesized *via* the method of Claisen–Schmidt condensation. The desired chalcone derivatives 7a–d and 9a–f were obtained in good yields by reacting the 4-acetyl-5-thiophene-pyrazole with the appropriate heteroaryl aldehyde derivatives. The novel chalcones have undergone complete elemental analysis, ^1^H-NMR, ^13^C-NMR, mass spectrometry, and IR characterization. The three human cancer cell lines MCF7 (human Caucasian breast adenocarcinoma), PC3 (prostatic cancer) and PACA2 (pancreatic carcinoma) as well as the normal cell line BJ1 (normal skin fibroblasts) were tested *in vitro* for the anti-cancer properties of the newly synthesized chalcone derivatives. When compared to the reference medicine doxorubicin (IC_50_ = 52.1 μM), compound 9e showed the most promise derivative (IC_50_ = 27.6 μM) against PACA2 cells, while compound 7d demonstrated anticancer efficacy (IC_50_ = 42.6 μM against MCF7 cells compared to the reference drug doxorubicin (IC_50_ = 48 μM). Using breast and pancreatic cell lines, the gene expression, DNA damage, and DNA fragmentation percentages for compounds 7d and 9e were evaluated. Moreover, the molecular docking study of compounds 7d and 9e was assessed. The binding affinities of compound 9e toward P53 mutant Y220C was −22 kcal per mole, while those of compound 7d towards Bcl2 and CDK4 were −27.81 and −26.9 kcal per mole, respectively, compared to the standard values (−15.82, −33.96 and −29.9 kcal per mole).

## Abstract 2

A new series of pyrazole-based chalcones were synthesized and tested against MCF7, PC3, PACA2 and BJ1 as *in vitro* study. Gene expression, DNA damage, DNA fragmentation and molecular docking for were discussed.

## Graphical abstract

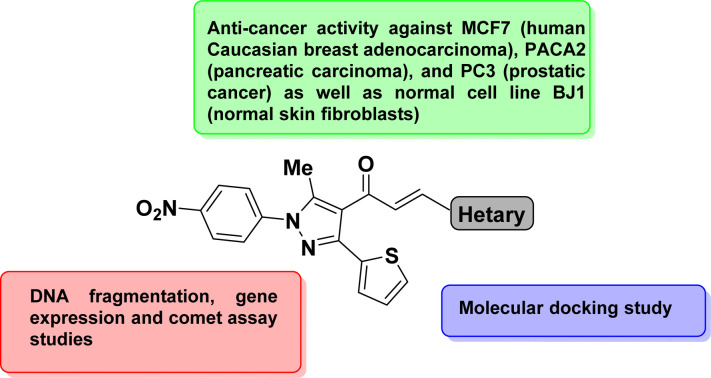

## 1 Introduction

Cancer cells arise when a cell ignores the rules governing cell division and starts to proliferate according to its own agenda. In 2040, there will likely be 28.4 million instances of cancer worldwide. According to the GLOBOCAN 2020 report, the primary cause of cancer incidence worldwide in 2020 is no longer lung cancer but rather breast cancer in women, with 2.3 million new cases expected, accounting for 11.7% of all cases of cancer and prostate cancer. Prostate cancer accounts for 7.3% of all cancer cases and is the second most common cancer in men. It is also the fifth largest cause of cancer-related death among males.^1,2^ Pancreatic cancer is the seventh greatest cause of cancer death in both sexes and, due to its poor prognosis, accounts for nearly as many deaths (466 000) as cases (496 000).^1^ Chemotherapeutic drugs are a standard treatment for the majority of malignancies, every patient responds differently to this kind of treatment, particularly when their tumors are inadequately diagnosed. The necessity for combination therapy and/or targeted medicines is indicated by tumor heterogeneity, the existence of cancer stem cells, and the adaptability of these cells.^3^ With multi-drug-resistant tumors continuously evolving, developing new drugs with enhanced efficacy is essential. The two main goals of cancer treatments are to destroy cancer cells (cytotoxic impact) and stop them from proliferating (cytostatic effect).^4^

The α,β-unsaturated variants of chalcones, which are secondary metabolites of plants, have a more stable thermodynamic *trans*-conformation among two aryl groups. Chalcones can be generated easily utilizing the Claisen–Schmidt condensation reaction, which is a commonly employed method to synthesize these compounds by condensation of carbonyl derivatives with the presence of a base.^5–7^ Numerous biological and pharmacological behavior, including anticancer, antibacterial, anti-proliferative, antifungal, antioxidant, and anti-inflammatory properties, have been linked to chalcones that incorporate heterocyclic scaffolds.^6,8,9^ In addition, it has been found that 3-(thiophen-2-yl)-1*H*-pyrazoles have promising biological implications.^7,10–15^ Cancer and death rates continue to increase dramatically worldwide. The growth of cancer is characterized by its rapidity and adaptability, which makes it difficult to find new therapies that will offer more potent therapeutic treatments due to drug resistance and side effects. In light of these results, and in keeping with our research interest in the synthesis of bioactive heterocycles,^2,16–23^ we were motivated to create a novel series of chalcones (7a–d and 9a–f) associated with heterocyclic moieties and assess their anti-tumor efficacy *in vitro* using different human cancer cell lines. The novel chalcones 7d and 9e proved potent and interesting cytotoxic effects against breast cancer cell lines (MCF7) and pancreatic cancer cell lines (PACA2), respectively compared with doxorubicin as a reference drug. Moreover the DNA fragmentation, comet assay and molecular docking studies were investigated for the most promising compounds (7d and 9e).

## 2 Results and discussion

### 2.1 Chemistry

**Scheme 1:**
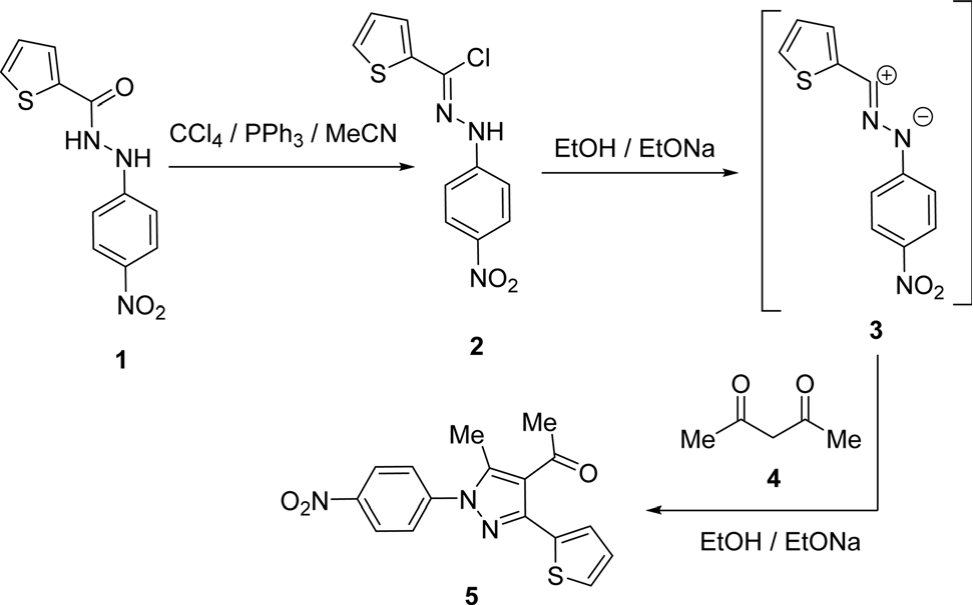
Synthesis of 1-(3-(thiophen-2-yl)-1*H*-pyrazol-4-yl)ethan-1-one (5).

1-(3-(Thiophen-2-yl)-1*H*-pyrazol-4-yl)ethan-1-one (5) was synthesized straightforwardly as reported in the literature^24^ and depicted in Scheme 1 and used as a suitable precursor to prepare the titled chalcone derivatives. Chlorination of thiophene-2-carbohydrazide (1) gave thiophene-2-carbohydrazonoyl chloride (2). The treatment of 2 with acetylacetone (4) in an ethanolic sodium ethoxide solution, 5 is produced *via* the intermediacy of nitrilimine (3) (Scheme 1).

**Scheme 2:**
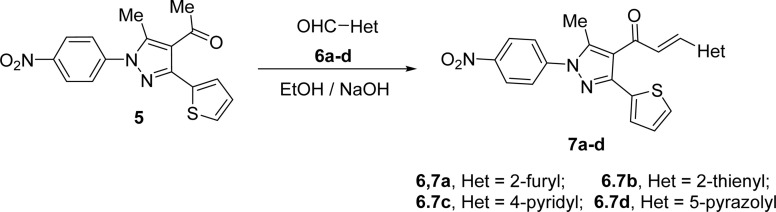
Synthesis of chalcone derivatives 7a–d.

**Fig. 1:**
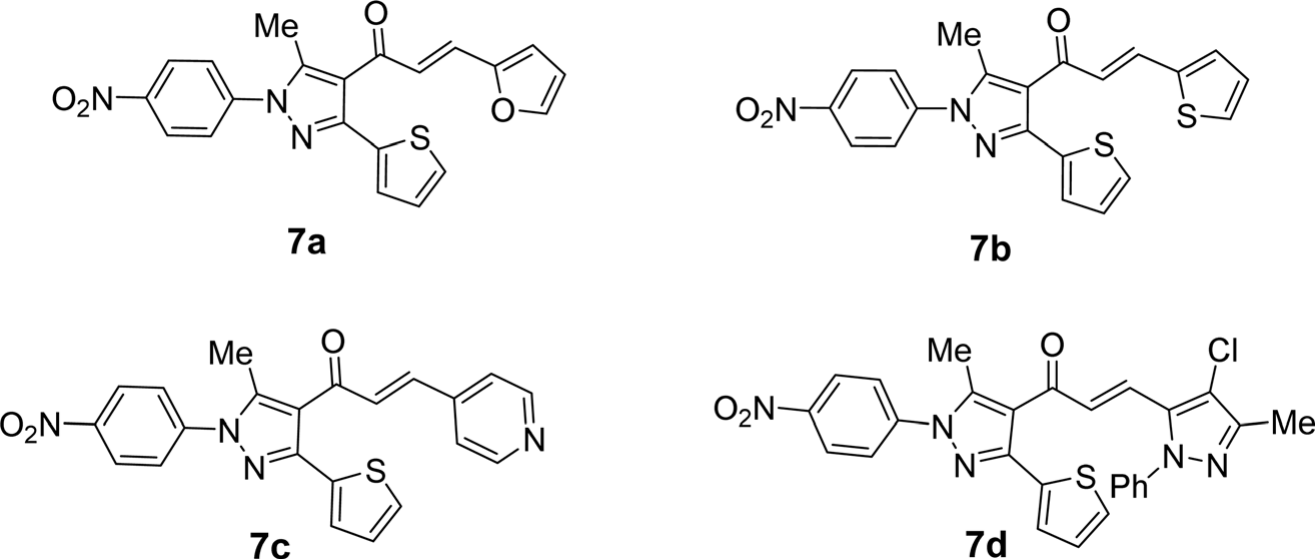
Chemical structures of the targeted chalcone derivatives 7a–d.

4-Acetyl-3-(thiophen-2-yl)-1*H*-pyrazole (5) in ethanol and the presence of NaOH solution underwent Claisen–Schmidt condensation with equimolar amounts of heteroaldehydes 6a–d (specifically, furan-2-carbaldehyde 6a, thiophene-2-carbaldehyde 6b, isonicotinaldehyde 6c, 4-chloro-3-methyl-1-phenyl-1*H*-pyrazole-5-carbaldehyde 6d) afforded the corresponding chalcone derivatives, 3-(thiophen-2-yl)pyrazolyl-chalcones (7a–d) as depicted in Schemes 2 and Fig. 1.

Based on their spectrum data, the end products of the synthesized chalcones' chemical structures were established. ^1^H NMR (300 MHz, CDCl_3_) spectrum of 7d as a representative example for series of 7a–d showed the presence of two singlet signals at *δ* 2.18 and 2.68 (ppm) for the methyl groups. The protons of the vinyl group were observed at *δ* 6.95 (ppm) as a doublet with coupling constant *J* = 15.9 (Hz) indicting *trans* configuration. The other aromatic protons appear at their expected position at *δ* 7.27–7.79 (ppm). Due to the symmetry in some carbons in compound 7d, 23 carbon' signals in the ^13^C-NMR spectrum were recorded and the carbon of the carbonyl group was observed at *δ* 186.6 (ppm).

**Scheme 3:**
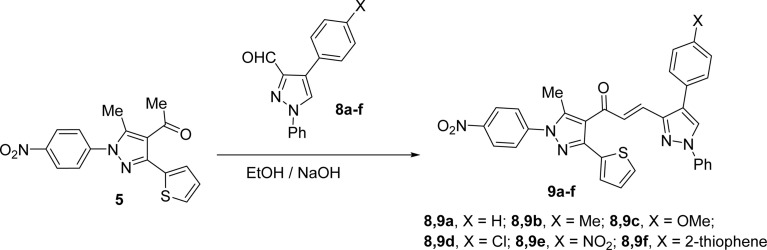
Synthesis of chalcone derivatives 9a–f.

**Fig. 2:**
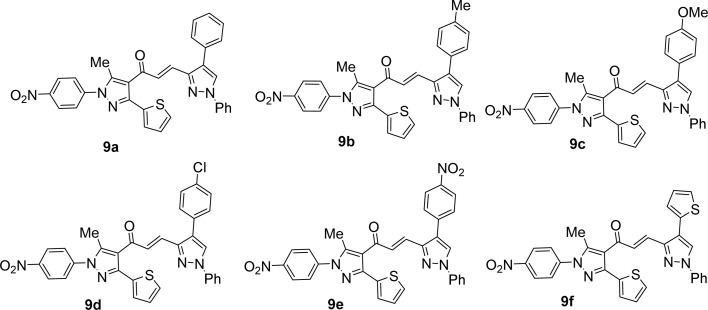
Chemical structures of the targeted chalcone derivatives 9a–f.

Likewise, the treatment of 5 with pyrazole-2-carboxaldehyde derivatives (8a–f) (specifically, 1,4-diphenyl-1*H*-pyrazole-3-carbaldehyde 8a, 1-phenyl-4-(*p*-tolyl)-1*H*-pyrazole-3-carbaldehyde 8b, 4-(4-methoxyphenyl)-1-phenyl-1*H*-pyrazole-3-carbaldehyde 8c, 4-(4-chlorophenyl)-1-phenyl-1*H*-pyrazole-3-carbaldehyde 8d, 4-(4-nitrophenyl)-1-phenyl-1*H*-pyrazole-3-carbaldehyde 8e, 1-phenyl-4-(thiophen-2-yl)-1*H*-pyrazole-3-carbaldehyde 8f) produced the targeted chalcone derivatives (9a–f) in good to excellent yield (Schemes 3 and Fig. 2).

### 2.2 Anti-cancer activity

The obtained results were summarized in Table 1 and showed that compound 7d possesses anticancer activities in the range of 70–80% on MCF7, PC3, and PACA2 cell lines, while compounds 7a and 9f exhibited specific anticancer activity on MCF7 cell line 63.5 and 80.4%, respectively. Compound 9e showed specific anticancer activity on the PACA2 cell line 100%. Compounds 7a, 9e and 7d exhibited limited inhibition activity on BJ1 cell lines (11.2, 5.6, and 12.3% respectively). The other compounds showed low activity against MCF7, PC3, and PACA2 cell lines as presented in Table 1. To find their IC_50_, the most efficacious compounds (7a, 9e, and 7d) underwent secondary screening.

**Table 1:** (%) Mortality of cancer and normal cell lines at 100 μg ml^−1^

Compound	MCF7	PACA2	PC3	BJ1
7a	**63.5 ± 0.11**	17.3 ± 0.13	3.8 ± 0.65	11.2 ± 0.48
7b	45.3 ± 0.98	32.5 ± 0.74	4.2 ± 0.73	—
7c	**100% up to 6.25 μg ml^−^** ^**1**^	**100% up to 6.25 μg ml^−^** ^**1**^	**100% up to 6.25 μg ml^−^** ^**1**^	52.3 ± 0.76
7d	**71.2 ± 0.14**	**80.3 ± 019**	**70.6 ± 0.97**	**12.3 ± 0.23**
9a	42.3 ± 0.21	**50.3 ± 0.42**	4.5 ± 0.26	—
9b	44.5 ± 0.74	39.5 ± 0.91	27.2 ± 0.31	—
9c	25.2 ± 0.41	19.8 ± 0.68	2.9 ± 0.57	—
9d	44.2 ± 0.33	**54.5 ± 0.84**	3.5 ± 0.92	—
9e	14.2 ± 0.37	**100**	5.3 ± 0.94	**5.6 ± 0.55**
9f	**80.4 ± 0.25**	16.5 ± 0.35	4.5 ± 0.23	—
Doxorubicin	100	100	100	100

According to IC_50_ values, compound 7d had been found to be a promising chemical against the MCF7 cell line, with an IC_50_ value of 42.6 μM when compared to the reference drug doxorubicin (IC_50_ = 48.0 μM) (Table 2). Additionally, compound 9e showed the highest efficacy against the PACA2 cell line, with an IC_50_ of 27.6 μM compared to the reference drug doxorubicin (IC_50_ = 52.1 μM). Cancer can be treated in one of three ways: surgery, chemotherapy, or radiation. Chemotherapy, such as doxorubicin, is frequently given prior to surgery. The principal dose-limiting adverse effect of these chemicals is cardiotoxicity, which can result in heart failure in extreme situations.^25^ Thus, new therapeutic medications are required to counteract the negative effects of present treatments. We effectively synthesized a novel series of pyrazolyl-chalcone derivatives as powerful anticancer drugs and tested their *in vitro* cytotoxicity against three different human cancer cell lines. The expression levels of the apoptotic genes P53, BID, and CCND1 were investigated in PACA2 9e-treated cells, as well as the anti-apoptotic genes Bcl2, CDK4, and p21 in MCF7 7d-treated cells.

**Table 2:** IC_50_ (μM) for the most promising compounds

Compound	MCF7	PACA2	PC3	BJ1
7a	72.3 ± 0.34	—	—	—
7b	—	—	—	—
7c	—	—	—	86.2 ± 0.22
7d	**42.6 ± 0.71**	51.9 ± 0.29	66.9 ± 0.98	—
9a	—	—	—	—
9b	—	—	—	—
9c	—	—	—	—
9d	—	—	—	—
9e	59.3 ± 0.85	**27.6 ± 0.33**	—	—
9f	—	—	—	—
Doxorubicin	48.0 ± 0.74	52.1 ± 0.64	43.8 ± 0.24	13.6 ± 0.28

#### 2.2.1 Gene expression in the breast cell line

**Fig. 3:**
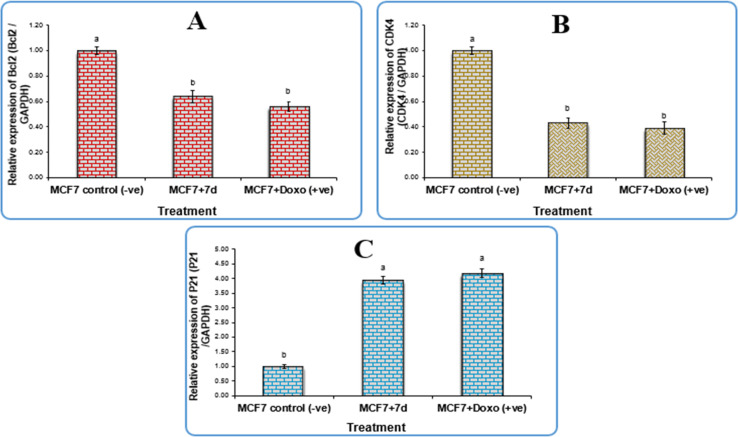
Gene expression of (a) BCL2 gene, (b) CDK4 gene, and (c) P21 gene in breast cancer cell lines treated with 7d compound. Data are presented as mean ± SEM. ^a,b,c^ mean values within tissue with different superscript letters were significantly different (*p* < 0.05).

The anti-apoptotic gene Bcl-2 (B-cell lymphoma 2), p21 (cyclin kinase inhibitor), CDK4 (cyclin-dependent kinase 4), and GAPDH (glyceraldehyde-3-phosphate dehydrogenase) were used as negative controls for gene expression study in the breast cancer cell line (MCF-7). The results revealed that cells treated with newly synthesised compound 7d and those treated with doxorubicin downregulated the expression levels of Bcl-2 and CDK4 significantly compared to the negative control, with fold change values of 0.64 and 0.43, respectively, but there were no significant differences (*P* > 0.05) between 7d and doxorubicin. Although the expression levels of the p21 gene were elevated greatly in cells treated with 7d compound and those treated with doxorubicin compared to the negative control with a fold change value of 3.95, there were no significant differences (*P* > 0.05) between 7d, and doxorubicin as shown in Fig. 3.

According to the qPCR results, which are displayed in Table 3, compound 7d successfully upregulated the p21 gene expression with a fold change value of 3.95. This indicates that compound 7d may be responsible for cell cycle arrest because the p21 protein is a universal inhibitor of multiple cyclin-dependent kinases, which pauses the G1 to S and G2 to M phases of the cell cycle.^26^ Conversely, it had a down-regulatory effect on the expression level of the antiapoptotic gene Bcl-2 and CDK4, with fold change values of 0.64 and 0.43, respectively. Mammary gland tumours require CDK4 in order to form.^27^ Tumorigenesis has been shown to be significantly impacted by downregulation of the CDK4/6-cyclin D/INK4/pRB/E2F axis, or its promoters.^28^

**Table 3:** The relative expression levels of Bcl2, CDK4 and P21 genes in MCF7 cells after the treatment with IC_50_ conc. of compound 7d, untreated A549 cells were used as a negative control

Gene expression (fold change/GAPDH) sample			
Sample	Bcl-2	CDK4	P21
7d	0.64	0.43	3.95
MCF7 +ve control	0.56	0.39	4.18
MCF7 −ve control	1	1	1

Bcl-2 is an essential component in preventing apoptosis by preventing the release of cyt-c from the mitochondria, and as such, it has been recognized as a possible target for establishing of innovative anti-tumor treatments.^29^ As a result, 7d may be employed as a Bcl-2 and CDK4 inhibitor to cause apoptosis in the therapy of breast cancer. Chalcones have been shown in earlier investigations to induce apoptosis by upregulating the expressions of Bcl-2 and p53 in several cancer cell lines.^2,18,30^

#### 2.2.2 Gene expression in the pancreatic cell line

**Fig. 4:**
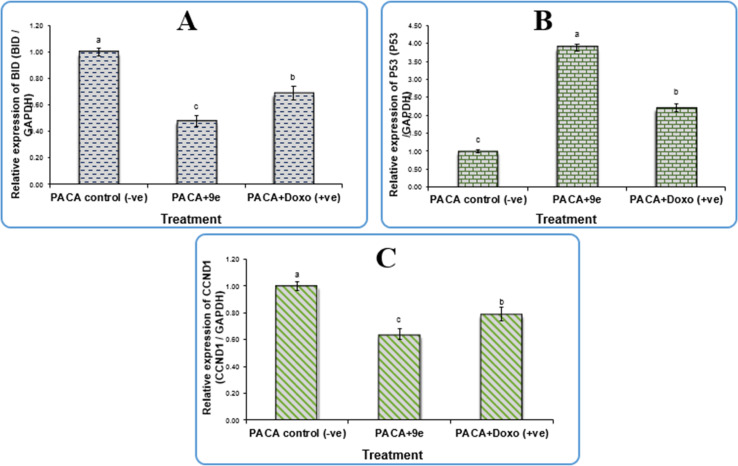
Gene expression of (A) BID gene, (B) P53 gene, and (C) CCND1 gene in pancreatic cancer cell lines treated with 9e compound. Data are presented as mean ± SEM. ^a,b,c^ mean values within tissue with different superscript letters were significantly different (*p* < 0.05).

Gene expression analysis in pancreatic cancer cell line (PACA2) was done using BID: BH3 interacting-domain death agonist, CCND1: cyclin D1, p53: tumor suppressor protein p53, and GAPDH: glyceraldehyde-3-phosphate dehydrogenase. The newly synthesized compound 9e and doxorubicin significantly downregulated mRNA levels of BID and CCND1 in the PACA2 cancer cell line compared to the negative control, with fold change values of 0.48 and 0.64, respectively. Also, there were significant differences between 9e and doxorubicin (*P* < 0.01). However, the treatment of compound 9e and doxorubicin upregulated the expression level of p53 when compared with a negative control with a fold change value of 3.89, there were also significant differences between 9e and doxorubicin (*P* < 0.01) as shown in Fig. 4.

In terms of qPCR data, our findings shown in Table 4 revealed that compound 9e upregulated p53 expression levels by a factor of 3.89, which is at the heart of the cell's tumor suppressive mechanism. Conversely, 9e downregulated the expression levels of BID and CCND1 with fold change values of 0.48 and 0.64, respectively. The primary cyclin involved in the cell's transition from the G1 to S phase, cyclin D1 (CCND1), is a critical modulator of cell cycle progression and is essential to the etiology of cancer.^31^ A member of the pro-apoptotic Bcl-2 family, Bid activates the intrinsic mechanism of apoptosis by activating Bak and Bax directly, allowing cytochrome c to be released from the mitochondria, and triggering the activation of downstream caspases, which in turn causes cell apoptosis.^32^ Our results confirm that the 9e compound induced apoptosis and cell cycle arrest and thus can be used as a promising antitumor therapeutic drug.

**Table 4:** The relative expression levels of P53, BID and CCND1 genes in PACA2 cells after the treatment with IC_50_ conc. of compound 9e, untreated A549 cells were used as a negative control

Gene expression (fold change/GAPDH) sample			
Sample	P53	BID	CCND1
9e	3.89	0.48	0.64
PACA +ve control	2.21	0.69	0.79
PACA −ve control	1	1	1

#### 2.2.3 Measurement of DNA fragmentation in the breast cancer cell line

**Fig. 5:**
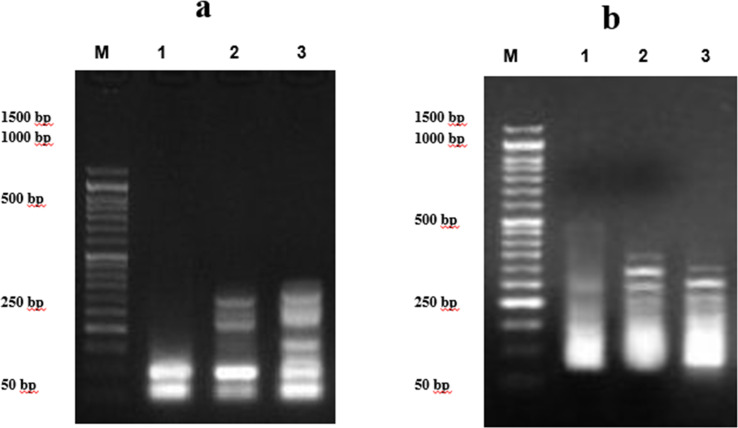
DNA fragmentation was detected by agarose gel electrophoresis. (a) Pancreatic cancer cell line treated with 9e. Lane 1 shows the negative cancer cell line; lane 2 shows the efficiency 9e in cancer cell line; and lane 3 shows cancer cell lines treated with doxorubicin. (b) Breast cancer cell line treated with 7d. Lane 1 shows the negative cancer cell line; lane 2 shows cancer cell line treated with 7d; and lane 3 shows cancer cell lines treated with doxorubicin. M represents the DNA marker.

The negative control group showed a substantial decrease in DNA fragmentation rates (8.6 ± 0.36%) compared to those treated with 7d compound and doxorubicin (25.2 ± 0.65%). In MCF7 cell lines treated with compound 7d, DNA fragmentation rates increased significantly (*P* < 0.001) compared to the negative control (21.8 ± 0.94%). Furthermore, MCF7-7d showed the highest rate of DNA fragmentation compared to doxorubicin-treated cells (Table 5 and Fig. 5a).

**Table 5:** DNA fragmentation was detected in MCF7 cell lines treated with 7d and doxorubicin^a^

Treatment	DNA fragmentation (%) M ± SEM	Change	Inhibition (%)
7d	21.8 ± 0.94^a^	13.2	20.48
MCF7 +ve control	25.2 ± 0.65^a^	16.6	23.67
MCF7 −ve control	8.6 ± 0.36^b^	0	0

a NB: means with different superscripts (^a^ and ^b^) between treatments in the same column are significantly different at *P* < 0.05.

#### 2.2.4 Measurement of DNA fragmentation in the pancreatic cancer cell line

Table 6 shows the rate of DNA fragmentation in the pancreatic cell line (PACA2). The negative control had significantly lower (*P* < 0.01) DNA fragmentation rates (8.3 ± 0.31%) than those treated with doxorubicin (21.5 ± 0.46%) and compound 9e. PACA2 cell lines treated with compound 9e showed considerably higher DNA fragmentation rates (24.2 ± 0.57) compared to the negative control (*P* < 0.001). Furthermore, Fig. 5b shows that PACA2-9e had the greatest rates of DNA fragmentation compared to doxorubicin-treated cells.

**Table 6:** DNA fragmentation was detected in PACA cell lines treated with 9e and doxorubicin^a^

Treatment	DNA fragmentation (%) M ± SEM	Change	Inhibition (%)
PACA (−ve) control	8.3 ± 0.31^b^	0	0
9e	24.2 ± 0.57^a^	15.9	24.45
PACA (+ve) control	21.5 ± 0.46^a^	13.2	17.94

a NB: means with different superscripts (^a^ and ^b^) between treatments in the same column are significantly different at *P* < 0.05.

We demonstrated the capability of chalcone derivatives to induce DNA damage. Compounds 9e and 7d significantly increased DNA damage in PACA2, and MCF7 human cancer cell lines, as indicated by DNA fragmentation tests. Similar results have been reported for chalcone derivatives, which lowered the long-term survival of HepG2 cancer cells owing to DNA fragmentation.^33^ Furthermore, chalcone derivatives totally destroyed the genomic DNA of HCT1.^7,20,21,33,34^ Similar conclusions have been observed for pyrazolyl-chalcones which markedly elevated the DNA fragmentation values (p 0.01) in the treated lung and liver cell line samples in contrast to the negative control.^15^ Chalcone derivatives have the potential for damaging DNA by binding DNA strands through interactions between aromatic ring stacking and van der Waal forces. Molecular docking study demonstrating that chalcones are linked to a DNA dodecamer with multiple hydrogen bonds provide evidence that the unsaturated carbonyl system in chalcone compounds allows greater electrostatic interactions between the hydrogen and DNA bases.^35^

#### 2.2.5 DNA damage in breast cancer cell lines

**Fig. 6:**
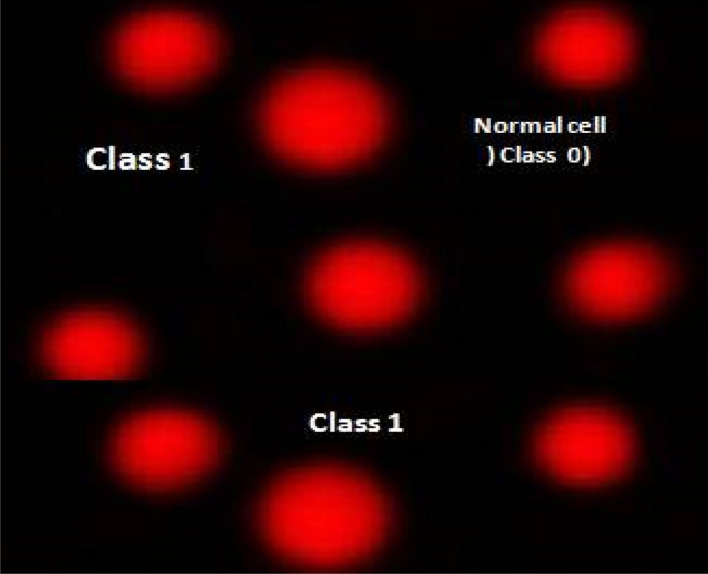
Visual score of normal DNA (class 0) using comet assay in breast cancer cell lines.

**Fig. 7:**
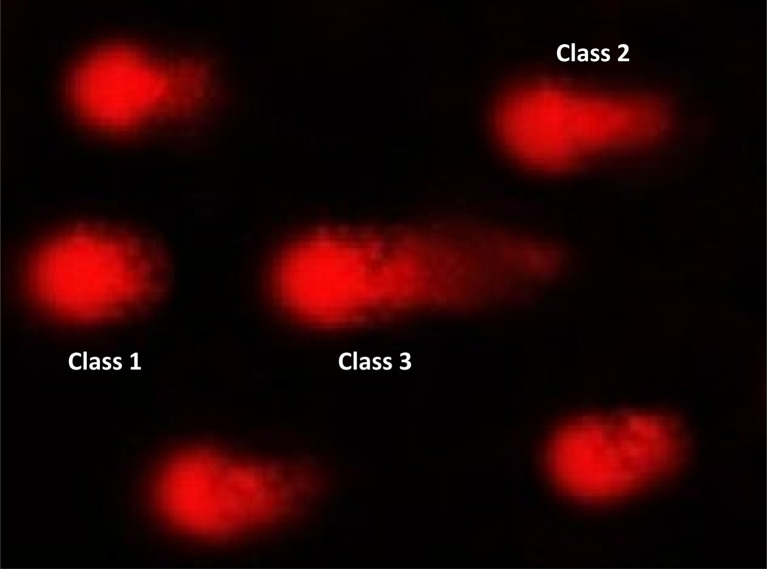
Visual score of DNA damage (classes 1, 2 and 3) using comet assay in breast cancer cell lines exposed to 7d.

Table 7, Fig. 6 and 7 illustrate how the comet assay for 7d was used to assess the DNA damage in breast cancer cell lines. Negative breast cancer cell lines showed a substantial reduction (*P* < 0.05) in DNA damage (9.63 ± 0.64). However, the 7d-treated breast cancer cell line sample showed considerably higher DNA damage values (25.931.08) compared to the negative control (9.630.64). Dox treatment resulted in considerably higher DNA damage values (21.980.91) in the positive breast cancer cell line compared to the negative control (9.630.64).

**Table 7:** Visual score of DNA damage in breast cancer cell lines treated with 7d

Treatment	No. of samples	No. of cells		Class^b^				DNA damaged cells% (mean ± SEM)
		Analyzed^a^	Comets	0	1	2	3	
MCF-7 (−ve)	4	405	39	366	26	9	4	9.63 ± 0.64^c^
MCF-7 + 7d	4	405	105	300	30	41	34	25.93 ± 1.08^a^
MCF-7 + Dox	4	405	89	316	27	33	29	21.98 ± 0.91^b^

a Number of cells examined per a group.

b Class 0 = no tail; 1 = tail length < diameter of nucleus; 2 = tail length between 1× and 2× the diameter of nucleus; and 3 = tail length > 2× the diameter of nucleus. Means with different superscripts (^a,b,c^) between groups in the same treatment are significantly different at *P* < 0.05. Data are presented as mean ± SEM.

#### 2.2.6 DNA damage in pancreatic cancer cell lines

**Fig. 8:**
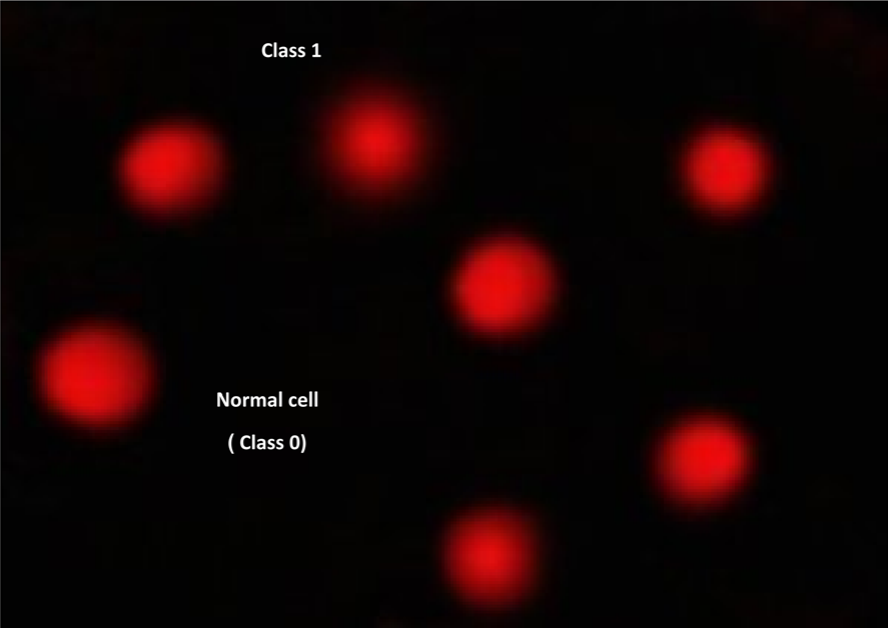
Visual score of normal DNA (class 0) and DNA damage (class 1) using comet assay in pancreatic cancer cell lines.

**Fig. 9:**
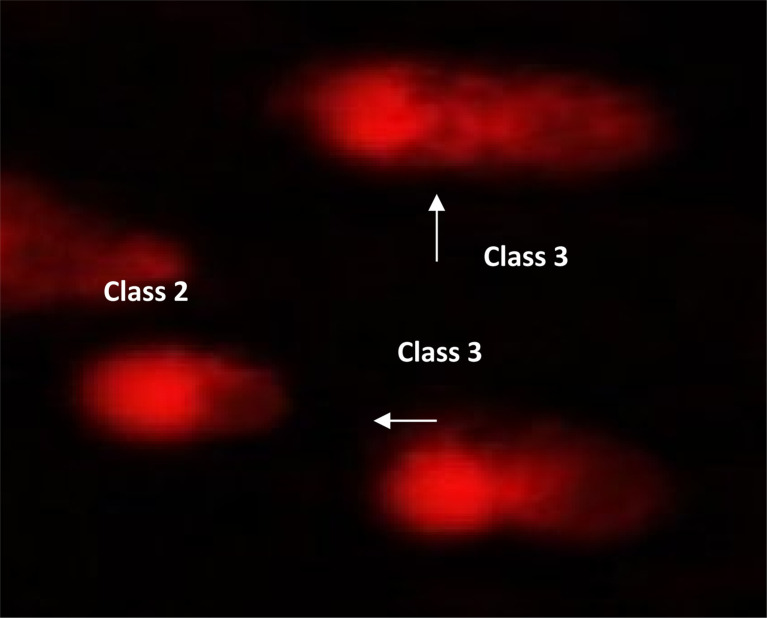
Visual score of DNA damage (classes 1, 2 and 3) using comet assay in pancreatic cancer cell lines exposed to 9e.

The DNA damage in pancreatic cancer cell lines was measured using the comet test for 9e, as indicated in Table 8, Fig. 8 and 9. Negative pancreatic cancer cell lines showed a substantial reduction (*P* < 0.05) in DNA damage levels (10.12 ± 0.85). However, the DNA damage values were considerably enhanced (*P* < 0.01) in the 9e-treated pancreatic cancer cell line (28.40 ± 1.04) and Dox-treated pancreatic cancer cell line (26.17 ± 1.38) compared to the negative control (10.12 ± 0.85).

**Table 8:** Visual score of DNA damage in pancreatic cancer cell lines treated with 9e

Treatment	No of samples	No. of cells		Class^b^				DNA damaged cells% (mean ± SEM)
		Analyzed^a^	Comets	0	1	2	3	
Paca2 (−ve)	4	405	41	364	29	9	3	10.12 ± 0.85^b^
Paca2 + 9e	4	405	115	290	35	37	43	28.40 ± 1.04^a^
Paca2 + Dox	4	405	106	299	38	32	36	26.17 ± 1.38^a^

a Number of cells examined per a group.

b Class 0 = no tail; 1 = tail length < diameter of nucleus; 2 = tail length between 1× and 2× the diameter of nucleus; and 3 = tail length > 2× the diameter of nucleus. Means with different superscripts (^a,b^) between groups in the same treatment are significantly different at *P* < 0.05. Data are presented as mean ± SEM.

### 2.3 Molecular docking study

Molecular docking is an excellent approach for predicting binding sites and the kind of interaction between interacting molecules. Compounds 7d and 9e have been selected for further molecular investigation because they showed the most promising cytotoxic activity against PACA2 and MCF7, respectively. Molecular docking investigations were carried out to establish the binding affinity of compound 9e to P53 mutant Y220C and compound 7d to anti-apoptotic Bcl2 and CDK4. The root mean squared deviation (RMSD) for P53 mutant Y220C, Bcl2, and CDK4 (PDB ID: 5O1H, 6QGG, and 1GIJ), respectively were 0.72, 2.4, and 1.8, indicating that docking findings were highly precise. The binding affinities of compound 9e toward P53 mutant Y220C was −22 kcal per mole while that of compound 7d towards Bcl2 and CDK4 were −27.81 and −26.9 kcal per mole respectively which were comparable to the standard values (−15.82, −33.96 and −29.9 kcal per mole).

**Fig. 10:**
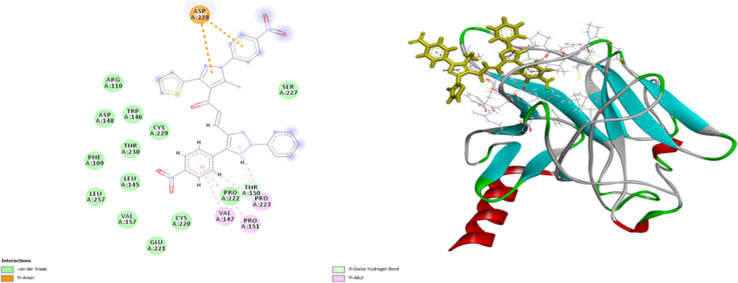
2D and 3D docking of compound 9e with P53 mutant Y220C.

**Fig. 11:**
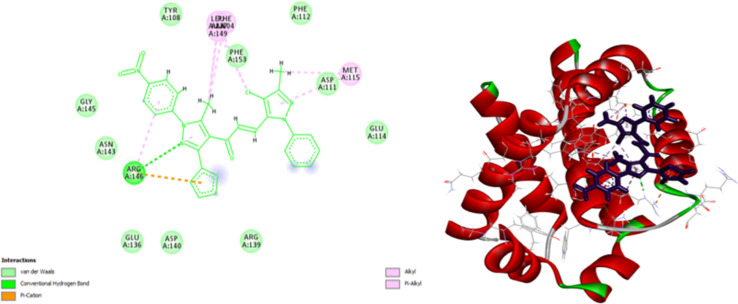
2D and 3D docking of compound 7d with BCL2.

As shown in Fig. 10, compound 9e reactivated p53 mutant Y220C and revealed a high docking score through seven interactions. Two pi-anion hydrophobic interactions were seen between the benzene ring of nitrobenzene moiety and ASP 228 with a bond distance of 5.58 Å and another one between imidazole moiety and ASP 228 with a bond distance of 4.46 Å. In addition to four pi-Alkyl hydrophobic bonds and the interacting amino acid residues were VAL 147, PRO 223 and PRO 151. Besides a pi–donor hydrogen bond with amino acid residues THR 150 with a bond distance of 5.39 Å. It was observed that compound 7d achieved a high docking score based on its interaction with protein Bcl2 *via* ten interactions, a pi–cation interaction between thiophene moiety and ARG 146 (5.21 Å), a conventional hydrogen bond between imidazole moiety and ARG 146 (4 Å), and the remaining eight interactions were alkyl and pi–alkyl interactions with (ARG 146, MET 115, LEU 137, PHEA 104 and ALA 104) amino acid residues (Fig. 11).

**Fig. 12:**
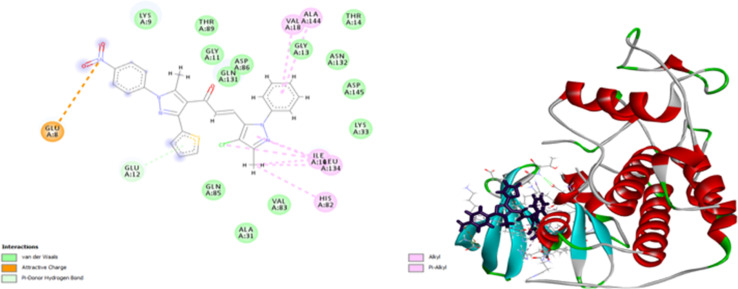
2D and 3D docking of compound 7d with CDK4.

Compound 7d inhibits CDK4 *via* ten interactions as shown in Fig. 11. These interactions include a pi donor hydrogen bond between GLU 12 and thiophene moiety (5.88 Å), an ionic bond between GLU 8 and nitrogen atom of nitrophenol moiety (5.28 Å).^36–40^ In addition to three alkyl interactions between methyl-chloro-imidazole moiety ILE 10 and LEU 134 amino acid residues (5.03, 5.04, 5.18 Å). Five pi–alkyl interactions with VALA 18, ALA 144, ILE 10, LEU 134, HIS 82 (5.80, 6.49, 4.87, 5.89, 4.85 Å) (Fig. 12). Since all docked poses with the lowest binding energy have the highest affinity, they are thought to be the best-docked conformations. So, from the above results, we could assume the activating effect of compound 9e on P53 mutant Y220C and the inhibitory effect of compound 7d against Bcl2 anti-apoptotic protein and CDK4. This assumption was confirmed in the subsequent gene expression assay.

#### 2.3.1 ADMET analysis

The ADMET studies of the two promising compounds were carried out using SwissADME web-based software.^41^ The leading novel chalcone molecules were identified based on their drug-like properties. Using the pkCSM (https://biosig.lab.uq.edu.au/pkcsm/prediction)^42^ online tool, the ADMET properties of chalcone derivatives 7d and 9e were calculated and presented in Table 9. The drug's distribution and absorption are greatly influenced by its water solubility (log *S*); medications are deemed highly soluble when their log *S* values are more than 4.^43^ The calculated log *S* values of compounds 7d and 9e revealed that they are poorly soluble (−10.47 and −9.06).

**Table 9:** *In silico* ADMET properties of the newly synthesized compounds 7d and 9e

Physicochemical properties		7d	9e
Adsorption	Water solubility (log mol L^−1^)	−5.737	−3.431
	Caco2 permeability (log Papp in 10^−6^ cm s^−1^)	0.789	−0.545
	Human intestinal absorption (% absorbed)	100	100
	Skin permeability (log *K*_p_)	−2.735	−2.735
	P-glycoprotein substrate (yes/no)	No	No
	P-glycoprotein I inhibitor (yes/no)	Yes	Yes
	P-glycoprotein II inhibitor (yes/no)	Yes	Yes
Distribution	Fraction unbound (human) (Fu)	0.316	0.382
	BBB permeability (log BB)	−1.401	−1.806
	CNS permeability (log PS)	−1.595	−1.662
Metabolism	CYP3A4 substrate (yes/no)	Yes	Yes
	CYP2C19 inhibitor (yes/no)	Yes	No
	CYP2C9 inhibitor (yes/no)	Yes	No
	CYP3A4 inhibitor (yes/no)	Yes	No
Excretion	Total clearance (log ml min^−1^ kg^−1^)	0.32	0.162
Toxicity	AMES toxicity (yes/no)	No	Yes
	Max. tolerated dose (human) (log mg per kg per day)	0.586	0.448
	Oral rat acute toxicity (LD_50_) (mol kg^−1^)	2.519	2.734
	Oral rat chronic toxicity (LOAEL) (log mg per kg per bw per day)	−0.499	−1.717
	Hepatotoxicity (yes/no)	Yes	Yes
	Skin sensitisation (yes/no)	No	No
	*T. Pyriformis* toxicity (log μg L^−1^)	0.285	0.285
	Minnow toxicity (log mM)	−5.073	−7.873

An alternative metric for assessing oral absorption of freshly manufactured medicines is the permeability coefficient across Caco-2 cell monolayers (PCaco-2).^44^ It is considered that a medication has strong Caco2 permeability if its predictive value is higher than 0.90.^42^ Compound 9e is considered slightly permeable (−0.545) while compound 7d was found to be relatively Caco2 permeable (0.789), as presented in Table 9. The amount of a drug that is orally absorbed *via* the human small intestine is estimated using an intestinal absorption parameter. A drug with an absorption rate of less than 30%^42^ is regarded as poorly absorbed, Therefore, Table 9 indicates that the chalcone derivatives 7d and 9e that were examined are fully absorbed *via* the human SI. A crucial aspect of transdermal drug distribution linked to pharmacological activity is skin permeability (log *K*_p_). A drug with a log *K*_p_ value of more than −2.5 (ref. 45) is supposed to have low skin permeability. Compounds 7d and 9e exhibited low skin permeability as their value was −2.735. P-glycoprotein functions as a biological barrier by squeezing out xenobiotics of the cell. The parameter predicts whether a given drug is a substrate of Pgp or not. Compounds 7d and 9e act as P-glycoprotein I and II inhibitors. The fraction of the medication that will be unbound in plasma is predicted by the fraction unbound (Fu) parameter. Fu values of compounds 7d and 9e were 0.382 and 0.316. The blood–brain barrier (BBB) may protect the brain from chemical substances that could be hazardous. The values of blood–brain permeability (log BB) of compounds 7d and 9e were (−1.806 and −1.401) higher than (−1)^45^ indicating that synthesized compounds had poor brain distribution. Correspondingly, the blood–brain permeability surface area product (log PS) of 7d and 9e were (−1.662 and −1.595) indicating that the inability of compounds to penetrate the Central Nervous System (CNS) as reference value of a drug to penetrate the CNS more than (−3).^46^ P450 genes encode a superfamily of detoxifying enzymes called cytochromes P450, which are mostly located in the liver. They play a crucial role in drug metabolisms to foster excretion. If the IC_50_ for each cytochrome P450 isoform is less than 10 μM, the drug is termed a cytochrome P450 inhibitor.^45^ CYP3A4 is one of the main isoforms implied in drug metabolism. Compounds 7d and 9e are predicted to be CYP3A4 substrates. In addition to 7d acts as CYP2C9, CYP2C19 and CYP3A4 inhibitors Table 9. Drug clearance is assessed using the proportion constant (CLtot), which is generally composed of hepatic and renal clearance. CLtot parameter is crucial in setting rates of dose. The total clearance log(CLtot) of the synthesized compounds 7d and 9e (0.162 and 0.32 (mlmin^−1^kg)) is shown in Table 9. AMES test is an assay to determine carcinogens using mutagenicity in bacteria, positive result indicates that the substance is mutagenic and consequently potentially carcinogenic.^47^ Synthetic 9e compound tested negative, indicating that it is not mutagenic while 7d tested positive. The drug's hazardous dosage threshold in humans is ascertained using the maximum recommended tolerated dose (MRTD). A substance is deemed non-toxic if its MRTD value is less than or equal to 0.477 [log(mg per kg per day)], while values higher than that are deemed toxic.^48^ The MRTD value of 9e is 0.448 thus it is nontoxic while that of 7d is 0.586 therefore it is considered slightly toxic. Lethal dose (LD_50_) is the amount of an ingested drug that kills 50% of test animal models. The drug is counted safe if LD_50_ values higher than 2.5 mol kg^−1^.^49^ The synthesized compounds 7d and 9e have an LD_50_ value slightly are higher than 2.5 mol kg^−1^ as demonstrated in Table 9, making them conceivably safe. The lowest amount of a drug that has been tested with negative effects on an exposed population is known as the lowest observed adverse effect level (LOAEL), while the greatest amount of a drug at which no adverse effects are recorded is known as the no adverse effect level (NOAEL). According to LOAEL values anticipated in log mg kg^−1^ bw/day (Table 9), the two synthetic compounds 7d and 9e have no negative impacts at larger doses. Disordered liver function is linked to hepatoxicity, and the expected values for the two synthesized chalcone derivatives 7d and 9e are positive. Oppositely, they anticipated negative values for Skin Sensitization. Toxicity of protozoa “*Tetrahymena pyriformi*” is often used as a lethal endpoint. If the plGC_50_ value of a drug is greater than −0.50 log μg L^−1^, the drug will be considered as toxin. The synthesized compounds 7d and 9e are toxic to *T. pyriformis*. To study toxicity in vertebrate animals, Flathead minnow is used as a model. If the drug concentration needed to kill 50% of the minnow sample population log LC_50_ value is <−0.3, it is considered to have acute toxicity.^50^ Compounds 7d and 9e have Flathead minnow toxicity.

## 3 Materials and methods

### 3.1 Chemistry

Melting points were measured with a Stuart melting point apparatus and were uncorrected. The IR spectra were recorded using a FTIR Bruker-vector 22 spectrophotometer as KBr pellets. The ^1^H and ^13^C NMR spectra were recorded in CDCl_3_ as a solvent on a Varian Gemini NMR spectrometer at 300 MHz using TMS as an internal standard. Chemical shifts are reported as *δ* values in ppm. Mass spectra were recorded with a Shimadzu GCMS-QP-1000 EX mass spectrometer in an EI (70 eV) model. The elemental analyses were performed at the Microanalytical Center, Cairo University. The 1-(5-methyl-1-(4-nitrophenyl)-3-(thiophen-2-yl)-1*H*-pyrazol-4-yl)ethan-1-one 5 (ref. 15 and 24) were prepared using the reported procedures.

### 3.2 Synthesis of 1-(5-methyl-1-(4-nitrophenyl)-3-(thiophen-2-yl)-1*H*-pyrazol-4-yl)prop-2-en-1-one derivatives (7a–d)

To a stirred mixture of 1-(5-methyl-1-(4-nitrophenyl)-3-(thiophen-2-yl)-1*H*-pyrazol-4-yl)ethan-1-one 5 (0.01 mol) and equimolar amounts of heteroaldehydes 6a–d (0.01 mol) in ethanol (30 ml), sodium hydroxide solution 20% was added and the reaction mixture was stirred for 4 h at room temperature and left overnight. The resulting solid product that precipitated was filtered, washed with water and crystallized from a suitable solvent to give the corresponding 1-(5-methyl-1-(4-nitrophenyl)-3-(thiophen-2-yl)-1*H*-pyrazol-4-yl)prop-2-en-1-one derivatives (7a–d).

#### 3.2.1 3-(Furan-2-yl)-1-(5-methyl-1-(4-nitrophenyl)-3-(thiophen-2-yl)-1*H*-pyrazol-4-yl)prop-2-en-1-one (7a)

Yellow solid, m.p. 110–112 °C, yield (82%). ^1^H NMR (300 MHz, CDCl_3_) *δ* (ppm) 2.61 (s, 3H, CH_3_), 6.46–6.47 (m, 1H, Ar–H), 6.64 (d, 1H, Ar–H, *J* = 3.3 Hz), 6.85 (d, 1H, vinyl-H, *J* = 15.3 Hz), 7.08 (m, 1H, Ar–H), 7.36–7.48 (m, 4H, Ar–H + vinyl-H), 7.76 (d, 2H, Ar–H, *J* = 9.3 Hz), 8.39 (d, 2H, Ar–H, *J* = 9 Hz). ^13^C NMR (75 MHz, CDCl_3_) *δ* (ppm) 12.8, 112.5, 116.0, 121.5, 123.6, 124.7, 125.4, 126.8, 127.5, 128.7, 129.6, 133.4, 143.2, 143.5, 145.0, 146.8, 151.3, 187.4. Anal. calcd for C_21_H_15_N_3_O_4_S (405.43): C, 62.21; H, 3.73; N, 10.36. Found: C, 62.36; H, 3.77; N, 10.42.

#### 3.2.2 1-(5-Methyl-1-(4-nitrophenyl)-3-(thiophen-2-yl)-1*H*-pyrazol-4-yl)-3-(thiophen-2-yl)prop-2-en-1-one (7b)

Yellow solid, m.p. 108–110 °C, yield (79%). ^1^H NMR (300 MHz, CDCl_3_) *δ* (ppm) 2.63 (s, 3H, CH_3_), 6.76 (d, 1H, vinyl-H, *J* = 15.3 Hz), 7.03–7.12 (m, 2H, Ar–H), 7.22–7.43 (m, 4H, Ar–H + vinyl-H), 7.75–7.81 (m, 3H, Ar–H), 8.39 (d, 2H, Ar–H, *J* = 9 Hz). ^13^C NMR (75 MHz, CDCl_3_) *δ* (ppm) 12.9, 121.6, 123.6, 124.7, 124.9, 125.4, 127.1, 127.6, 128.3, 128.9, 129.0, 131.7, 133.3, 135.6, 140.0, 143.4, 143.7, 146.0, 147.0, 186.8. MS (EI, *m*/*z*, %): 420.11 (M^−^, 6). Anal. calcd for C_21_H_15_N_3_O_3_S_2_ (421.49): C, 59.84; H, 3.59; N, 9.97. Found: C, 59.99; H, 3.64; N, 10.04.

#### 3.2.3 1-(5-Methyl-1-(4-nitrophenyl)-3-(thiophen-2-yl)-1*H*-pyrazol-4-yl)-3-(pyridin-4-yl)prop-2-en-1-one (7c)

Yellow solid, m.p. 120–121 °C, yield (92%). ^1^H NMR (300 MHz, CDCl_3_) *δ* (ppm) 2.65 (s, 3H, CH_3_), 6.56–6.58 (m, 1H, Ar–H), 6.85 (d, 1H, Ar–H, *J* = 6 Hz), 7.02–7.07 (d, 1H, vinyl-H, *J* = 15 Hz), 7.25–7.26 (m, 2H, Ar–H), 7.53–7.58 (m, 2H, Ar–H + vinyl-H), 7.75–7.78 (d, 2H, Ar–H, *J* = 9 Hz), 8.40–8.43 (d, 2H, Ar–H, *J* = 9 Hz), 8.62 (d, 2H, Ar–H, *J* = 3.0 Hz). ^13^C NMR (75 MHz, CDCl_3_) *δ* 12.7, 111.2, 111.7, 121.7, 124.7, 125.6, 129.6, 139.5, 141.9, 143.2, 143.6, 143.9, 144.2, 145.8, 147.0, 149.4, 150.4, 186.3. MS (EI, *m*/*z*, %): 415.21 (M^−^, 100). Anal. calcd for C_22_H_16_N_4_O_3_S (416.46): C, 63.45; H, 3.87; N, 13.45. Found: C, 63.57; H, 3.95; N, 13.59.

#### 3.2.4 3-(4-Chloro-3-methyl-1-phenyl-1*H*-pyrazol-5-yl)-1-(5-methyl-1-(4-nitrophenyl)-3-(thiophen-2-yl)-1*H*-pyrazol-4-yl)prop-2-en-1-one (7d)

White solid, m.p. 154–156 °C, yield (78%). ^1^H NMR (300 MHz, CDCl_3_) *δ* (ppm) 2.18 (s, 3H, CH_3_), 2.68 (s, 3H, CH_3_), 6.95 (d, 1H, vinyl-H, *J* = 15.9 Hz), 7.11 (t, 1H, Ar–H, *J* = 3 Hz), 7.27–7.79 (m, 10H, Ar–H + vinyl-H), 8.41 (d, 2H, Ar–H, *J* = 9 Hz). ^13^C NMR (75 MHz, CDCl_3_) *δ* (ppm) 12.8, 13.8, 113.9, 121.8, 124.4, 124.6, 124.7, 125.3, 125.4, 127.1, 127.5, 128.5, 128.9, 129.2, 131.3, 133.3, 137.4, 143.3, 143.9, 146.8, 146.9, 149.8, 186.6. Anal. calcd for C_27_H_20_ClN_5_O_3_S (530.00): C, 61.19; H, 3.80; N, 13.21. Found: C, 61.28; H, 3.84; N, 13.26.

### 3.3 Synthesis of 1-(5-methyl-1-(4-nitrophenyl)-3-(thiophen-2-yl)-1*H*-pyrazol-4-yl)prop-2-en-1-one derivatives (9a–d)

To a stirred mixture of 1-(5-methyl-1-(4-nitrophenyl)-3-(thiophen-2-yl)-1*H*-pyrazol-4-yl)ethan-1-one 5 (0.01 mol) and equimolar amounts of pyrazole-2-carboxaldehyde derivatives (8a–f) (0.01 mol) in ethanol (30 ml), sodium hydroxide solution 20% was added and the reaction mixture was stirred for 4 h at room temperature and left overnight. The resulting solid product that precipitated was filtered, washed with water and crystallized from a suitable solvent to give the corresponding 1-(5-methyl-1-(4-nitrophenyl)-3-(thiophen-2-yl)-1*H*-pyrazol-4-yl)prop-2-en-1-one derivatives (9a–f).

#### 3.3.1 3-(1,4-Diphenyl-1*H*-pyrazol-3-yl)-1-(5-methyl-1-(4-nitrophenyl)-3-(thiophen-2-yl)-1*H*-pyrazol-4-yl)prop-2-en-1-one (9a)

White solid, m.p. 160–162 °C, yield (75%). ^1^H NMR (300 MHz, CDCl_3_) *δ* (ppm) 2.60 (s, 3H, CH_3_), 6.79 (d, 1H, vinyl-H, *J* = 15.9 Hz), 7.13–7.76 (m, 16H, Ar–H + vinyl-H), 7.99 (s, 1H, pyrazole-H5), 8.39 (d, 2H, Ar–H, *J* = 9 Hz). MS (EI, *m*/*z*, %): 559.53 (M^2+^, 10). Anal. calcd for C_32_H_23_N_5_O_3_S (557.63): C, 68.93; H, 4.16; N, 12.56. Found: C, 68.97; H, 4.22; N, 12.66.

#### 3.3.2 1-(5-Methyl-1-(4-nitrophenyl)-3-(thiophen-2-yl)-1*H*-pyrazol-4-yl)-3-(1-phenyl-4-(*p*-tolyl)-1*H*-pyrazol-3-yl)prop-2-en-1-one (9b)

Beige solid, m.p. 166–168 °C, yield (90%). ^1^H NMR (300 MHz, CDCl_3_) *δ* (ppm) 2.38 (s, 3H, CH_3_), 2.44 (s, 3H, CH_3_), 7.01–7.14 (m, 2H, Ar–H + vinyl-H), 7.27–7.64 (m, 10H, Ar–H + vinyl-H), 7.87–7.93 (m, 4H, Ar–H), 8.42 (d, 2H, Ar–H, *J* = 9 Hz), 9.01 (s, 1H, pyrazole-H5). ^13^C NMR (75 MHz, CDCl_3_) *δ* (ppm) 12.2, 20.8, 117.1, 117.5, 118.6, 120.2, 124.8, 125.5, 126.3, 127.1, 127.3, 127.7, 127.9, 128.2, 128.3, 128.8, 128.9, 129.3, 129.4, 129.6, 133.6, 135.1, 138.2, 138.8, 142.8, 143.2, 145.5, 146.4, 152.8, 188.2. Anal. calcd for C_33_H_25_N_5_O_3_S (571.66): C, 69.34; H, 4.41; N, 12.25. Found: C, 69.45; H, 4.54; N, 12.31.

#### 3.3.3 3-(4-(4-Methoxyphenyl)-1-phenyl-1*H*-pyrazol-3-yl)-1-(5-methyl-1-(4-nitrophenyl)-3-(thiophen-2-yl)-1*H*-pyrazol-4-yl)prop-2-en-1-one (9c)

Yellow solid, m.p. 180–182 °C, yield (89%). ^1^H NMR (300 MHz, CDCl_3_) *δ* (ppm) 2.59 (s, 3H, CH_3_), 3.91 (s, 3H, OCH_3_), 7.00–7.12 (m, 4H, Ar–H + vinyl-H), 7.37–7.64 (m, 8H, Ar–H + vinyl-H), 7.86–7.95 (m, 4H, Ar–H), 8.42 (d, 2H, Ar–H, *J* = 9 Hz), 9.05 (s, 1H, pyrazole-H5). ^13^C NMR (75 MHz, CDCl_3_) *δ* (ppm) 12.3, 55.2, 114.2, 117.0, 118.6, 120.3, 124.1, 124.8, 125.4, 126.0, 127.0, 127.2, 127.7, 127.9, 128.7, 129.6, 133.6, 134.9, 138.8, 142.8, 143.1, 145.6, 146.4, 152.6, 159.6, 188.0. Anal. calcd for C_33_H_25_N_5_O_4_S (587.65): C, 67.45; H, 4.29; N, 11.92. Found: C, 67.55; H, 4.32; N, 12.01.

#### 3.3.4 3-(4-(4-Chlorophenyl)-1-phenyl-1*H*-pyrazol-3-yl)-1-(5-methyl-1-(4-nitrophenyl)-3-(thiophen-2-yl)-1*H*-pyrazol-4-yl)prop-2-en-1-one (9d)

White solid, m.p. 152–154 °C, yield (85%). ^1^H NMR (300 MHz, CDCl_3_) *δ* (ppm) 2.50 (s, 3H, CH_3_), 7.00–7.14 (m, 2H, Ar–H + vinyl-H), 7.40–7.64 (m, 9H, Ar–H + vinyl-H), 7.87–7.94 (m, 5H, Ar–H), 8.42 (d, 2H, Ar–H, *J* = 9 Hz), 9.08 (s, 1H, pyrazole-H5). ^13^C NMR (75 MHz, CDCl_3_) *δ* (ppm) 12.4, 117.3, 118.8, 120.2, 124.5, 125.7, 126.3, 126.6, 127.4, 127.8, 128.1, 128.9, 129.3, 129.8, 130.0, 130.7, 133.5, 133.6, 134.4, 138.8, 143.1, 143.2, 145.6, 146.5, 151.5, 188.0. MS (EI, *m*/*z*, %): 594.41 (M^2+^, 3). Anal. calcd for C_32_H_22_ClN_5_O_3_S (592.07): C, 64.92; H, 3.75; N, 11.83. Found: C, 64.95; H, 3.88; N, 11.95.

#### 3.3.5 1-(5-Methyl-1-(4-nitrophenyl)-3-(thiophen-2-yl)-1*H*-pyrazol-4-yl)-3-(4-(4-nitrophenyl)-1-phenyl-1*H*-pyrazol-3-yl)prop-2-en-1-one (9e)

Yellow solid, m.p. 162–164 °C, yield (77%). ^1^H NMR (300 MHz, CDCl_3_) *δ* (ppm) 2.51 (s, 3H, CH_3_), 7.03–7.11 (m, 2H, Ar–H + vinyl-H), 7.41–7.64 (m, 8H, Ar–H + vinyl-H), 7.90–7.95 (m, 4H, Ar–H), 8.31–8.44 (m, 4H, Ar–H), 9.14 (s, 1H, pyrazole-H5). ^13^C NMR (75 MHz, CDCl_3_) *δ* (ppm) 12.4, 117.8, 118.8, 119.3, 120.2, 123.6, 124.0, 125.6, 127.0, 127.3, 127.5, 127.7, 128.2, 129.2, 129.5, 129.7, 133.5, 133.6, 138.3, 138.6, 143.1, 143.2, 145.6, 146.5, 147.3, 150.2, 187.6. MS (EI, *m*/*z*, %): 600 (M^2−^, 7). Anal. calcd for C_32_H_22_N_6_O_5_S (602.63): C, 63.78; H, 3.68; N, 13.95. Found: C, 63.82; H, 3.74; N, 14.00.

#### 3.3.6 1-(5-Methyl-1-(4-nitrophenyl)-3-(thiophen-2-yl)-1*H*-pyrazol-4-yl)-3-(1-phenyl-4-(thiophen-2-yl)-1*H*-pyrazol-3-yl)prop-2-en-1-one (9f)

Yellow solid, m.p. 118–120 °C, yield (84%). ^1^H NMR (300 MHz, CDCl_3_) *δ* (ppm) 2.53 (s, 3H, CH_3_), 7.08–7.19 (m, 4H, Ar–H + vinyl-H), 7.37–7.69 (m, 7H, Ar–H + vinyl-H), 7.85–7.95 (m, 4H, Ar–H), 8.43 (d, 2H, Ar–H, *J* = 9 Hz), 9.08 (s, 1H, pyrazole-H5). ^13^C NMR (75 MHz, CDCl_3_) *δ* (ppm) 12.3, 117.0, 118.7, 120.2, 124.9, 125.6, 126.9, 127.3, 127.4, 127.7, 128.0, 128.1, 128.9, 129.7, 133.2, 133.6, 134.1, 138.6, 142.8, 143.2, 145.6, 146.5, 146.8, 188.1. MS (EI, *m*/*z*, %): 563.94 (M, 27). Anal. calcd for C_30_H_21_N_5_O_3_S_2_ (563.65): C, 63.93; H, 3.76; N, 12.43. Found: C, 63.98; H, 3.77; N, 12.48.

### 3.4 Anti-cancer evaluation

The percentage of the cell viability treated with the newly synthesized compounds was evaluated using MTT assay (3-(4,5-dimethylthiazol-2-yl)-2,5-diphenyl tetrazolium bromide). All the subsequent steps were done in a sterile Laminar flow class II biosafety cabinet (Baker, SG406INT, Sanford, ME, USA). DMEM was used as a suspension medium for breast cancer (MCF7), prostate cancer (PC3), and pancreatic cancer (PACA2), while normal cell line (BJ1) was suspended in DMEM-F12 medium, a 1% antibiotic-antimycotic mixture (10 000 U ml^−1^ potassium penicillin, 10 000 μg ml^−1^ streptomycin sulfate, and 25 μg ml^−1^ amphotericin B), and 1% l-glutamine at 37 °C under 5% CO_2_.

Cells were cultured for 10 days and then seeded at a concentration of 10 × 10^4^ cells per well in fresh complete growth medium in 96-well microtiter plastic plates at 37 °C for 24 hours under 5% CO_2_ using a water-jacketed carbon dioxide incubator (Sheldon, TC2323). Media were aspirated, serum-free fresh medium was added, and cells were incubated either alone (negative control) or with different concentrations of the synthesized chalcone derivatives to a final concentration of (100–50–25–12.5–6.25–3.125–0.78 and 1.56 μg ml^−1^). After incubation for 48 hours, the medium was aspirated and 40 μl of MTT salt (2.5 μg ml^−1^) was added to each well and incubated for another four hours at 37 °C under 5% CO_2_. To stop the reaction and dissolve the formed crystals, 200 μl of 10% sodium dodecyl sulfate (SDS) in deionized water was added to each well and incubated overnight at 37 °C. Doxorubicin was used as a positive control of concentration 100 μg ml^−1^.^19–22,33^

The absorbance was then measured at 595 nm and a reference wavelength of 620 nm using a microplate multi-well reader (Bio-Rad Laboratories Inc., model 3350). Statistical significance was tested between the samples and the negative control (cells with vehicle) using an independent *t*-test using the SPSS 11 program. DMSO was the vehicle used for the dissolution of the synthesized chalcones, and its final concentration on the cells was less than 0.2%. The percentage of change in viability was calculated according to the formula: ((reading of extract/reading of negative control) − 1) × 100. A Probit analysis was carried out for the determination of IC_50_ using the SPSS 11 program.

### 3.5 Gene expression analysis

#### 3.5.1 RNA isolation and reverse transcription (RT) reaction

Total RNA was isolated from breast and pancreatic cancer cell lines according to the manufacturer's protocol using RNeasy Mini Kit (Qiagen) supplemented with the DNaseI (Qiagen) digestion step. To digest DNA residues, the isolated total RNA was treated with one unit of RQ1 RNAse-free DNAse (Invitrogen), re-suspended in DEPC-treated water, and quantified using a spectrophotometer at 260 nm. The purity of total RNA was assessed by the 260/280 nm ratio, which was between 1.8 and 2.1. Moreover, RNA integrity was assured by ethidium bromide-stain analysis of 28S and 18S bands by formaldehyde-containing agarose gel electrophoresis. Complete poly(A)+ RNA isolated from breast and pancreatic cell lines was reverse-transcribed into cDNA in a total volume of 20 μl using the RevertAidTM First Strand cDNA Synthesis Kit (Fermentas). An amount of total RNA (5 μg) was used with a master mix in a total of 20 μl in Eppendorf tubes. The master mix consisted of 50 mM MgCl_2_, 10× RT buffer (50 mM KCl; 10 mM Tris–HCl; pH 8.3), 10 mM of each dNTP, 50 μM oligo-dT primer, 20 IU ribonuclease inhibitor (50 kDa recombinant enzyme to inhibit RNase activity), and 50 IU MuLV reverse transcriptase. The mixture of each sample was centrifuged for 30 seconds at 1000 g and transferred to the thermocycler. The RT reaction was carried out at 25 °C for 10 minutes, followed by 1 hour at 42 °C, and completed with a denaturation step at 99 °C for 5 minutes. Subsequently, the reaction tubes containing RT preparations were flash-cooled in an ice chamber until used for cDNA amplification using a quantitative real-time polymerase chain reaction (qRT-PCR).

#### 3.5.2 Real-time quantitative PCR (qPCR)

Determination of the breast and pancreatic cell line cDNA copy number was carried out using the StepOne™ Real-Time PCR system from Applied Biosystems (Thermo Fisher Scientific). PCRs were set up in 25 μl reaction mixtures containing 12.5 μl of 1×SYBR^®^ Premix Ex TaqTM (TaKaRa, Biotech. Co. Ltd), 0.5 μl of 0.2 μM sense primer, 0.5 μl of 0.2 μM antisense primer, 6.5 μl of distilled water, and 5 μl of the cDNA template. The reaction program included three steps. The first step was performed at 95 °C for 3 minutes. The second step consisted of 40 cycles, in which each cycle was divided into three steps: (a) at 95 °C for 15 seconds; (b) at 55 °C for 30 seconds; and (c) at 72 °C for 30 seconds. The third step consisted of 71 cycles, which started at 60 °C and then increased by about 0.5 °C every 10 seconds up to 95 °C. At the end of each qRT-PCR, a melting curve analysis was performed at 95 °C to check the quality of the primers used. Each experiment included a distilled water control. Primers of the cancer-related genes were designed and are listed in Table 10. To check the quality of the used primers at the end of each qPCR, a melting curve analysis was performed at 95 °C. The relative quantification of the target to the reference was performed using the 2^−ΔΔCT^ method (Yang *et al.*, 2017).^51^

**Table 10:** Primer sequences used for real-time PCR of breast and pancreatic cancer cell lines^a^

Gene	Primer sequence	GenBank (accession no)
BID	F: GGCCTACCCTAGAGACATGG	CU012947.1
	R: TGGCTAAGCTCCTCACGTAG	
CCND1	F: GCATGTTCGTGGCCTCTAAG	NM_053056.3
	R: CGTGTTTGCGGATGATCTGT	
P53	F: TGGCCATCTACAAGCAGTCA	AB082923.1
	R: GGTACAGTCAGAGCCAACCT	
GAPDH	F: CCAAGGAGTAAGACCCCTGG	NM_001256799.3
	R: TGGTTGAGCACAGGGTACTT	
BCL2	F: CAAGTGTTCCGCGTGATTGA	KY098799.1
	R: CAGAGGAAAAGCAACGGGG	
CDK4	F: AGTGTGAGAGTCCCCAATGG	KR709914.1
	R: CCTTGATCTCCCGGTCAGTT	
P21	F: CCCAAGCTCTACCTTCCCAC	S67388.1
	R: CTGAGAGTCTCCAGGTCCAC	

a Abbreviations: BID: BH3 interacting-domain death agonist, CCND1: cyclin D1, p53: tumor suppressor protein p53, Bcl-2: B-cell lymphoma 2, p21: cyclin kinase inhibitor, CDK4: cyclin dependent kinase 4, and GAPDH: glyceraldehyde-3-phosphate dehydrogenase.

#### 3.5.3 DNA fragmentation assay

The DNA fragmentation assay in breast and pancreatic cancer cell lines was performed in accordance with the method of Helmy *et al.*, 2022 (ref. 15) with some modifications. Briefly, after 24 hours of exposure of breast and pancreatic cancer cell lines to the tested chalcone compounds in different Petri dishes (60 × 15 mm, Greiner), the cells were trypsinized, suspended, homogenized in 1 ml of medium, and centrifuged (10 min at 800 rpm). Low-molecular-weight genomic DNA was extracted as described in Yawata *et al.*, 1998.^52^ Approximately 1 × 10^6^ cells were plated and treated with the tested substances in various treatments. All the cells (including floating cells) were harvested by trypsinization and washed with Dulbecco's phosphate-buffered saline. Cells were lysed with the lysis buffer containing 10 mM Tris (pH 7.4), 150 mM NaCl, 5 mM EDTA, and 0.5% Triton X-100 for 30 minutes on ice. Lysates were vortexed and cleared by centrifugation at 10 000 g for 20 min. Fragmented DNA in the supernatant was extracted with an equal volume of a neutral phenol/chloroform/isoamyl alcohol mixture (25 : 24 : 1) and analyzed electrophoretically on 2% agarose gels containing 0.1 μg ml^−1^ ethidium bromide.

#### 3.5.4 Determination of the DNA damage using the comet assay

Olive *et al.* (1990) used pancreatic and breast cancer cell lines to measure the DNA damage using the comet test.^53^ Approximately 1.5 × 104 cells were imbedded in 0.75% low-gelling-temperature agarose after trypsin treatment to generate a single-cell suspension, which was then quickly pipetted onto a pre-coated microscope slide. Cell samples were lysed in 0.5% SDS, 30 mM EDTA, pH 8.0, for four hours at 50 °C. Samples were electrophoresed for 25 minutes at 0.6 V cm^−1^ after being rinsed overnight at room temperature in Tris/borate/EDTA buffer, pH 8.0. Propidium iodide was then added for staining. A fluorescent microscope equipped with a CCD camera was used to observe the slides, and 150 unique comet photos from each sample were examined to determine the tail moment, DNA content, and percentage of DNA in the tail. To find the fraction of cells with DNA damage that resemble comets in each sample, around 100 cells were analyzed. Based on perceived comet tail length migration and relative proportion of DNA in the nucleus, the non-overlapping cells were randomly chosen and visually assigned a score on an arbitrary scale of 0–3. Class 0 = no detectable DNA damage and no tail; class 1 = tail with a length less than the nucleus diameter; class 2 = tail with length between 1× and 2× the nuclear diameter; and class 3 = tail longer than 2× the nucleus diameter.^54^

#### 3.5.5 Molecular docking

Using (MOE) program 2009.10 version, molecular docking for compounds 9e and 7d were conducted in accordance with (Sroor *et al.*, 2023)^2,18^ to examine the ligand–protein interactions at the active sites of the P53 mutant Y220C, Bcl2 and CDK4 proteins. The structure of CDK4 has not been solved yet.^55^ synthesized a CDK4 mimic CDK2 protein by substituting the ATP binding pocket of CDK2 with that of CDK4 and keeping the other fragments of CDK2 the same.^56^ The X-ray crystallographic structures of the selected proteins were retrieved from the protein database (PDB) (https://www.rcsb.org/) (PDB ID: 5O1H, 6QGG and 1GIJ). The selected proteins were first prepared for modeling study where the standard ligand molecule was removed from the active site of the protein, the heavy atoms were kept fixed, and the hydrogen atoms were added to the whole protein structure to achieve optimization. The dock scoring in the MOE program was carried out using the London dG scoring function, and the upgrading was then performed using two unrelated refining techniques. It was optimized to set all parameters and charges to the MMFF94x force field, and the RMS gradient was adjusted at 0.01 kcal per mole, the RMS distance at 0.1 Å. At the final step, the ligand interaction (MOE) structure was saved as a PDB file to be visualized by the BIOVIA Discovery Studio V6.1.0.15350 program, where the newly synthesized compounds appeared to fit into the active domain of proteins in 2D and 3D states.^34^

#### 3.5.6 ADMET analysis

*In silico* techniques were used to inspect the most promising synthesized chalcone compounds (7d and 9e). The structures were drawn using PubChem to construct the isomeric SMILES format for these computations. The absorption, distribution, metabolism, excretion, and toxicity (ADMET) of the newly synthesized chalcone compounds were predicted using the free online tools SwissADME (http://www.swissadme.ch/) and pkCSM (http://biosig.unimerb.edu.au/pkcsm/).

## 4 Conclusion

In summary, a novel ten chalcone derivatives bearing pyrazole ring were prepared and assessed as anti-cancer molecules against normal cell line BJ1 and human cancer cell lines of MCF7, PACA2 and PC3. Compound 7d demonstrated anticancer activity with IC_50_ = 42.6 μM against MCF7 cells compared to the reference medication doxorubicin (IC_50_ = 48 μM), and compound 9e emerged as the most promising molecule with IC_50_ = 27.6 μM against PACA2 cells compared to the reference drug doxorubicin (IC_50_ = 52.1 μM). Using breast and pancreatic cell lines, the gene expression, DNA damage, and DNA fragmentation percentages for compounds 7d and 9e were evaluated. Moreover, the molecular docking study has been discussed.

## Author contributions

Norhan Yasser: anti-cancer activity, gene expression, DNA damage, molecular docking study. Farid Sroor: conceptualization, design, chemical reactions, methodology, writing – original draft, writing – review & editing, visualization, supervision, data curation, investigation. Haidan El-Shorbagy: supervision. Shaymaa Eissa: supervision. Hamdi M. Hassaneen: supervision, investigation. Ismail Abdelhamid: supervision, investigation, writing – original draft. All authors have seen and approved the submitted manuscript.

## Conflicts of interest

There are no financial or other relationships that might lead to a conflict of interest.

## Supplementary Material

RA-014-D4RA03375B-s001
