# Supplementary material for: Synthesis, anticancer evaluation of novel hybrid pyrazole-based chalcones, molecular docking, DNA fragmentation, and gene expression: in vitro studies
Source: RSC Adv. 2024 Jul 9;14(30):21859–73. doi: 10.1039/d4ra03375b (PMC11232109; doi:10.1039/d4ra03375b)
Supplement: RA-014-D4RA03375B-s001 [file RA-014-D4RA03375B-s001.pdf]

Electronic Supplementary Material (ESI) for RSC Advances.
This journal is © The Royal Society of Chemistry 2024

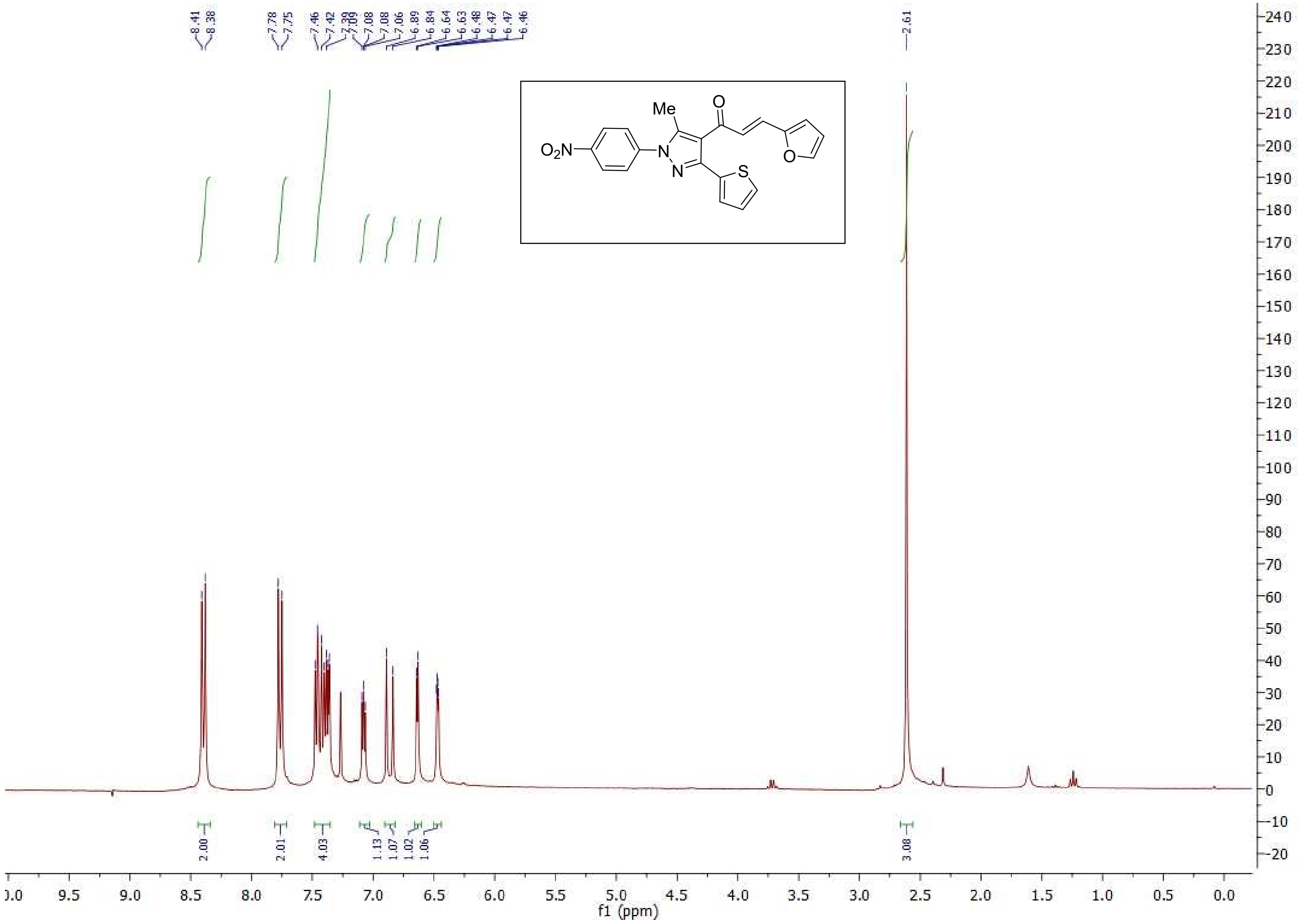

^{1}H-NMR spectrum of **7a**

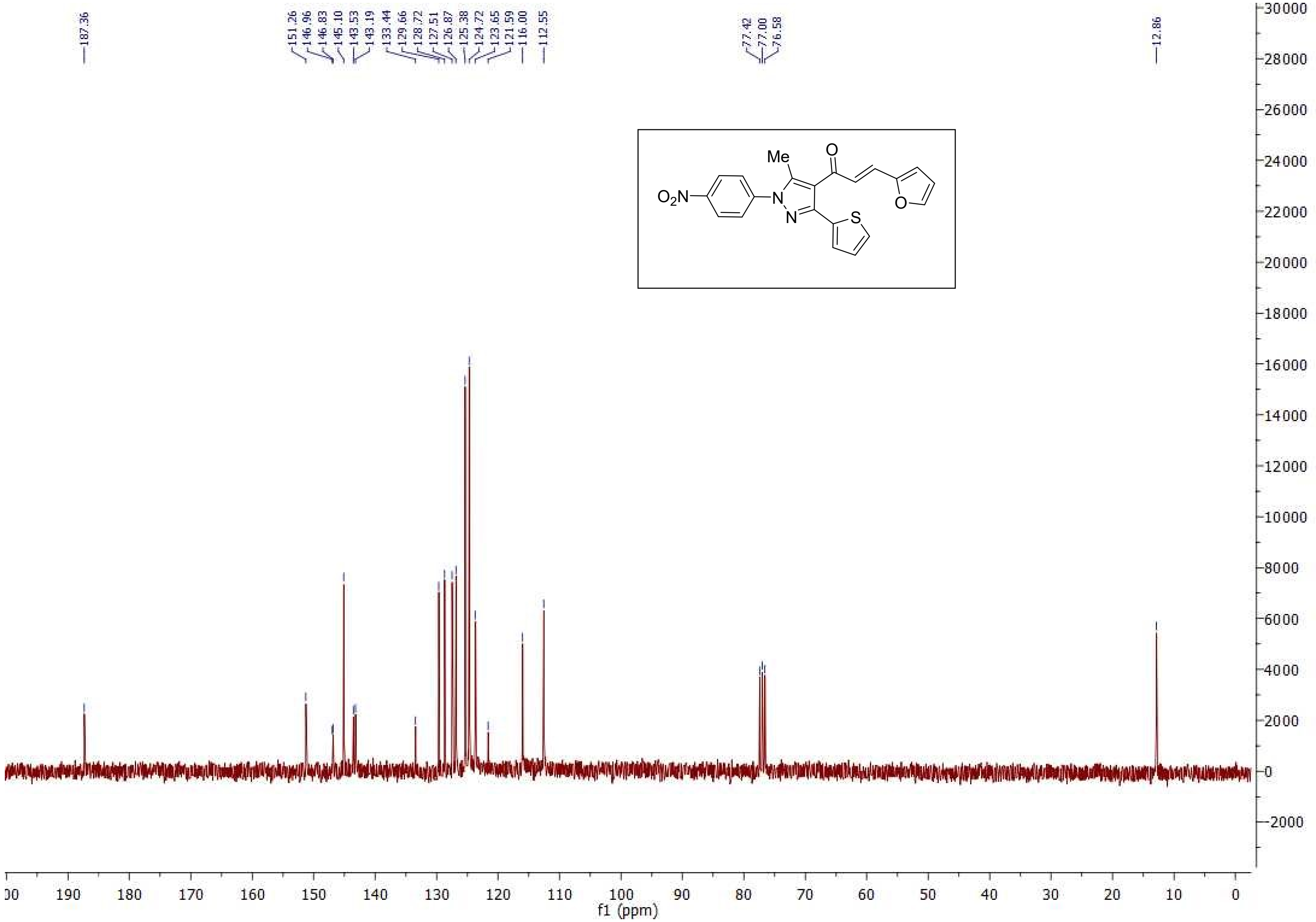

^{13}C-NMR spectrum of **7a**

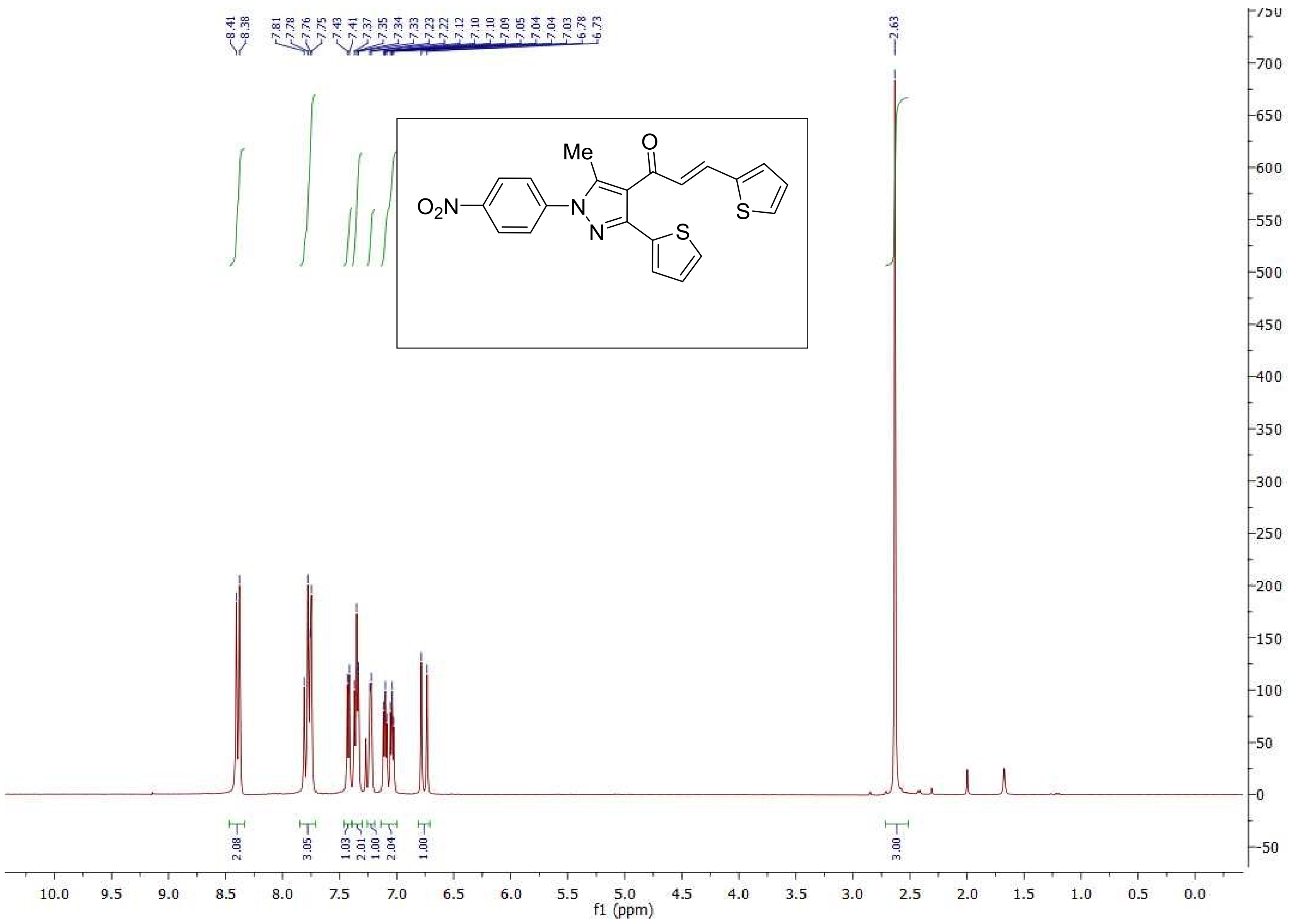

^{1}H-NMR spectrum of **7b**

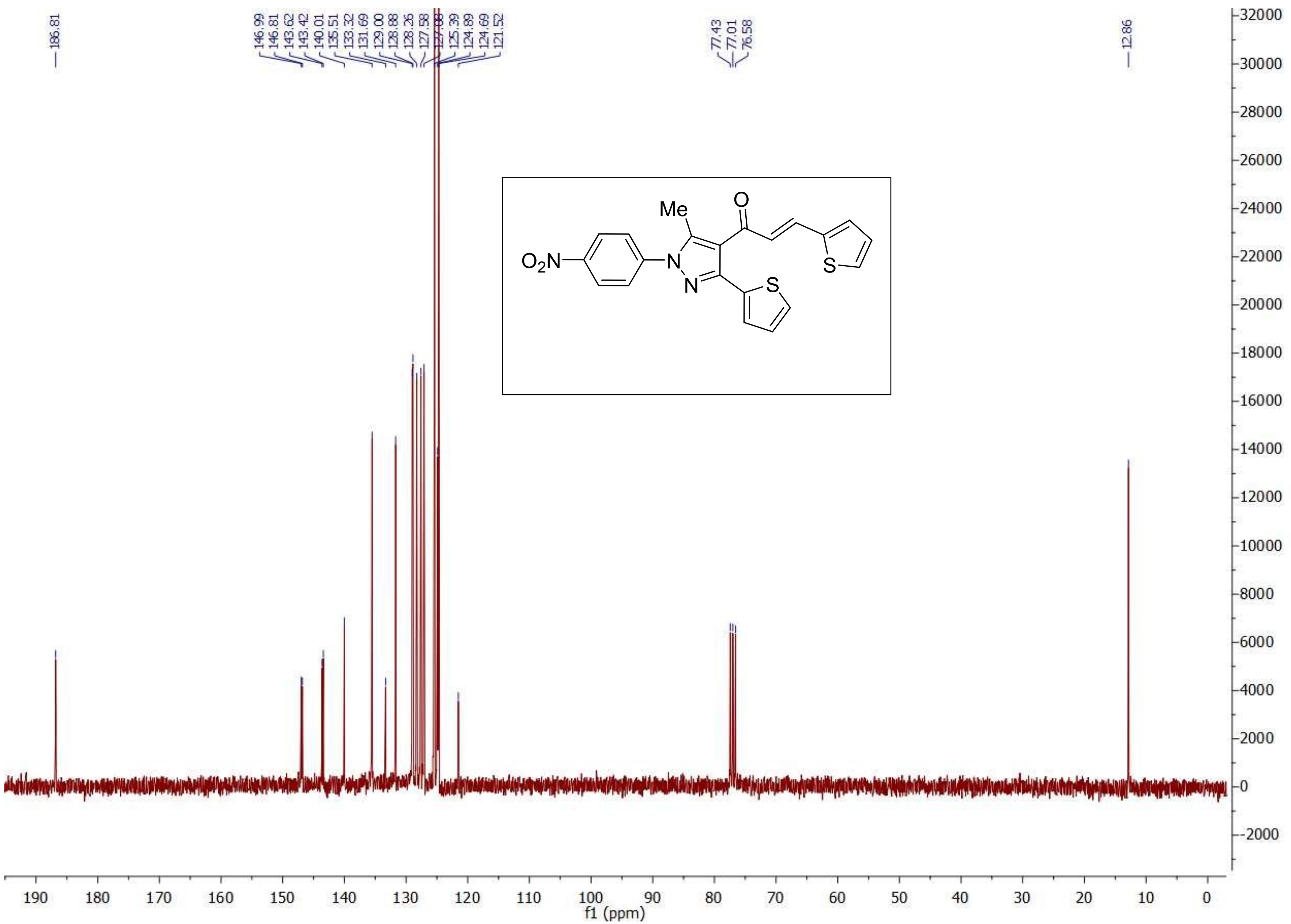

^{13}C-NMR spectrum of **7b**

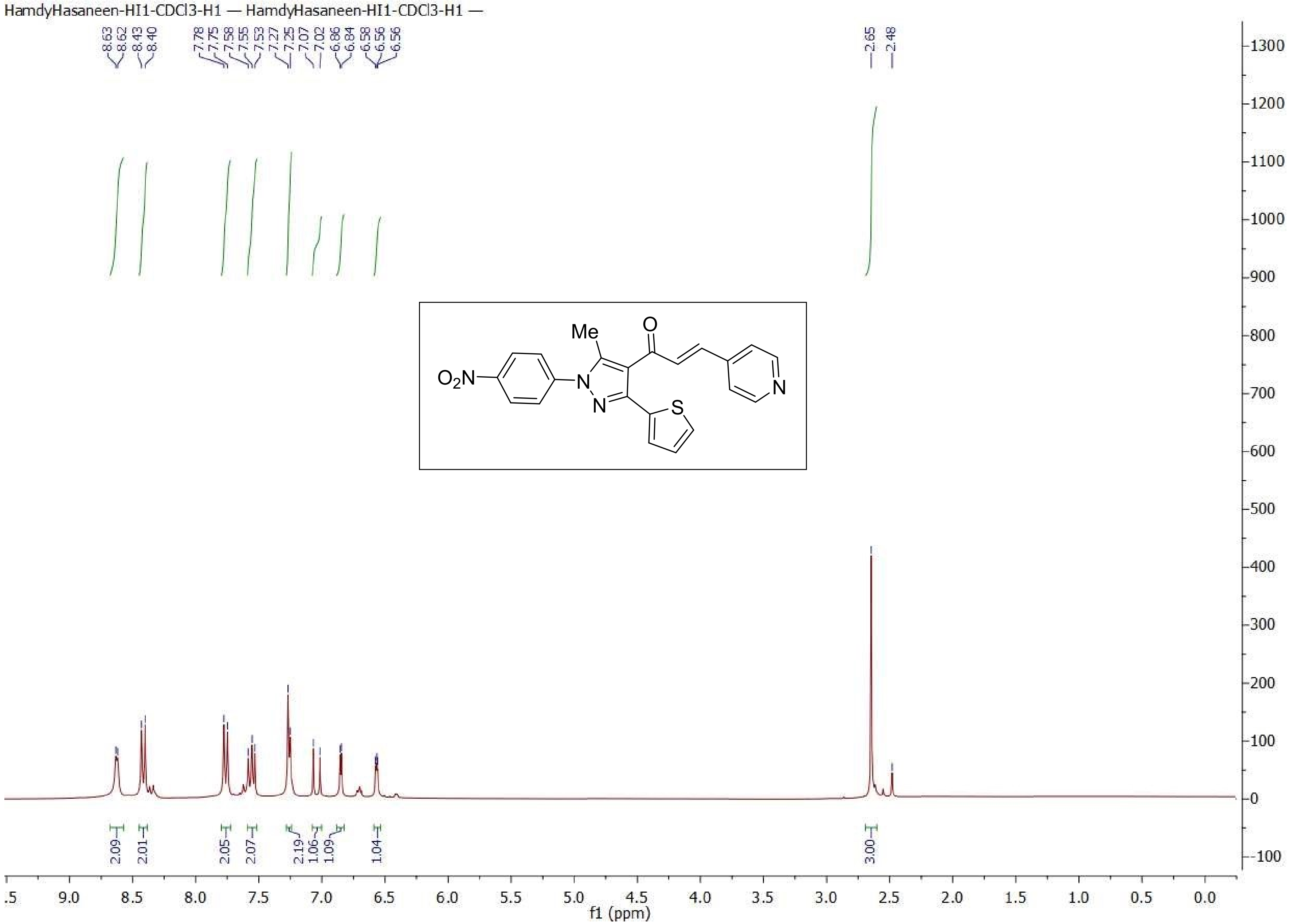

^{1}H-NMR spectrum of **7c**

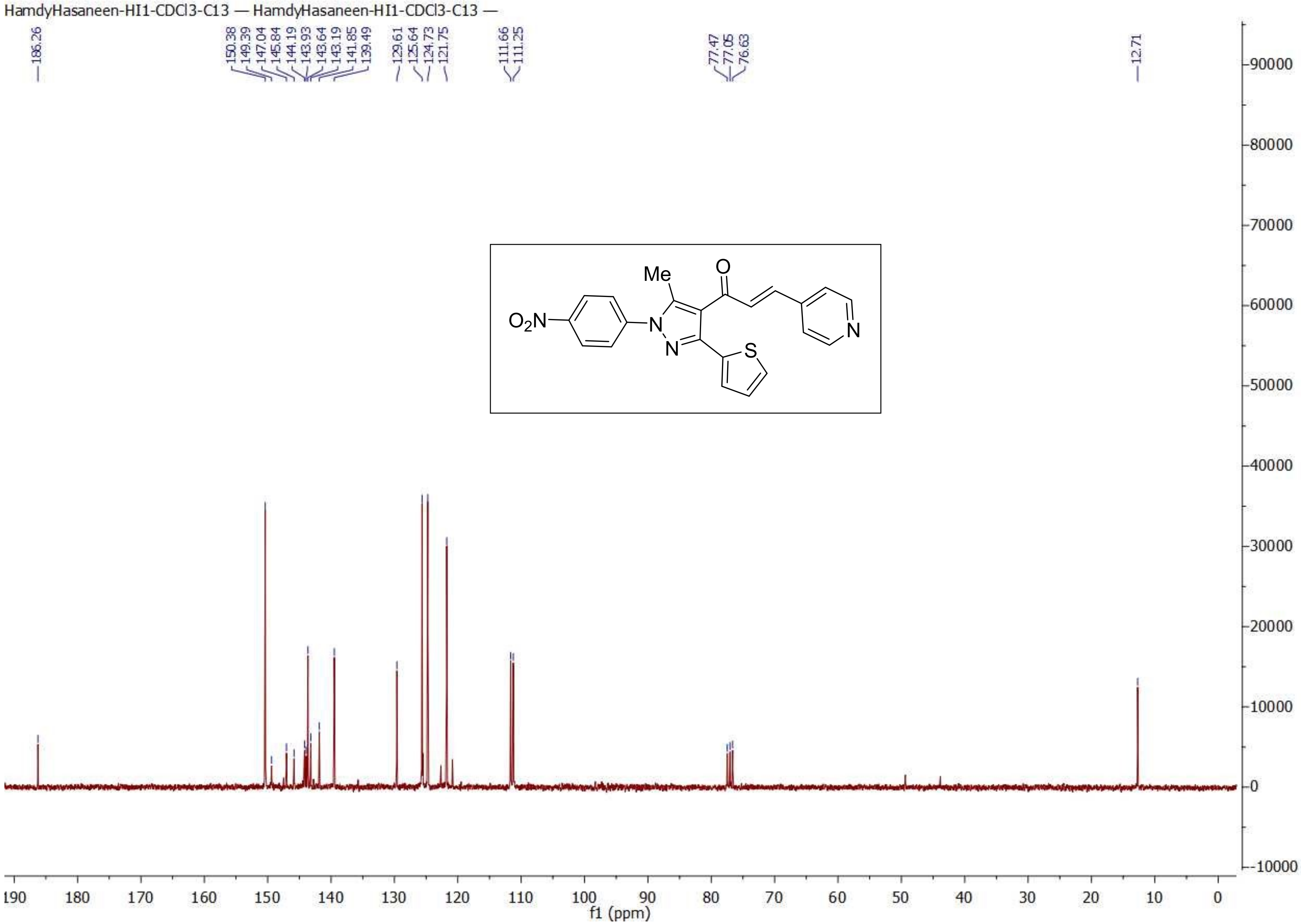

^{13}C-NMR spectrum of **7c**

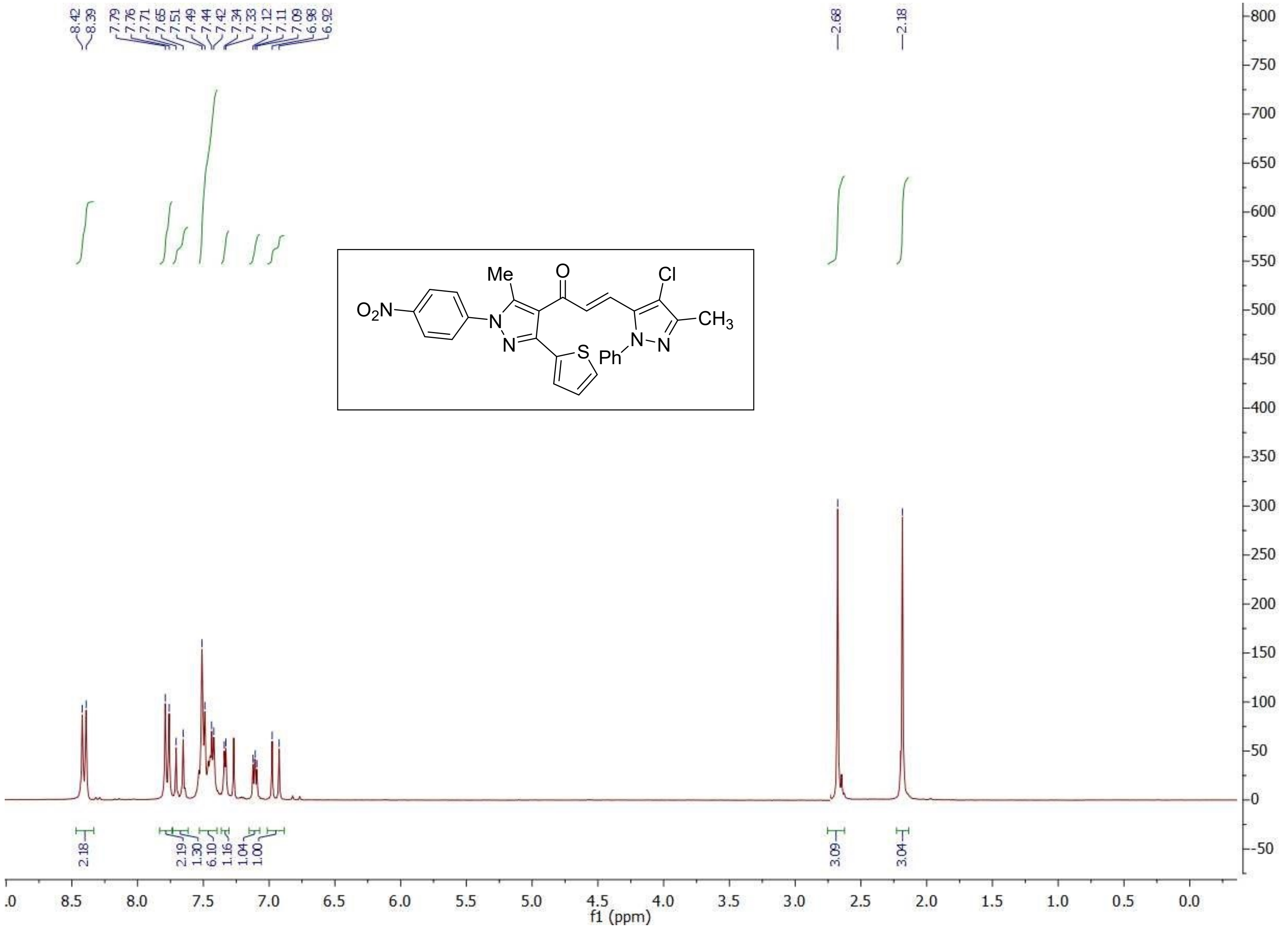

^{1}H-NMR spectrum of **7d**

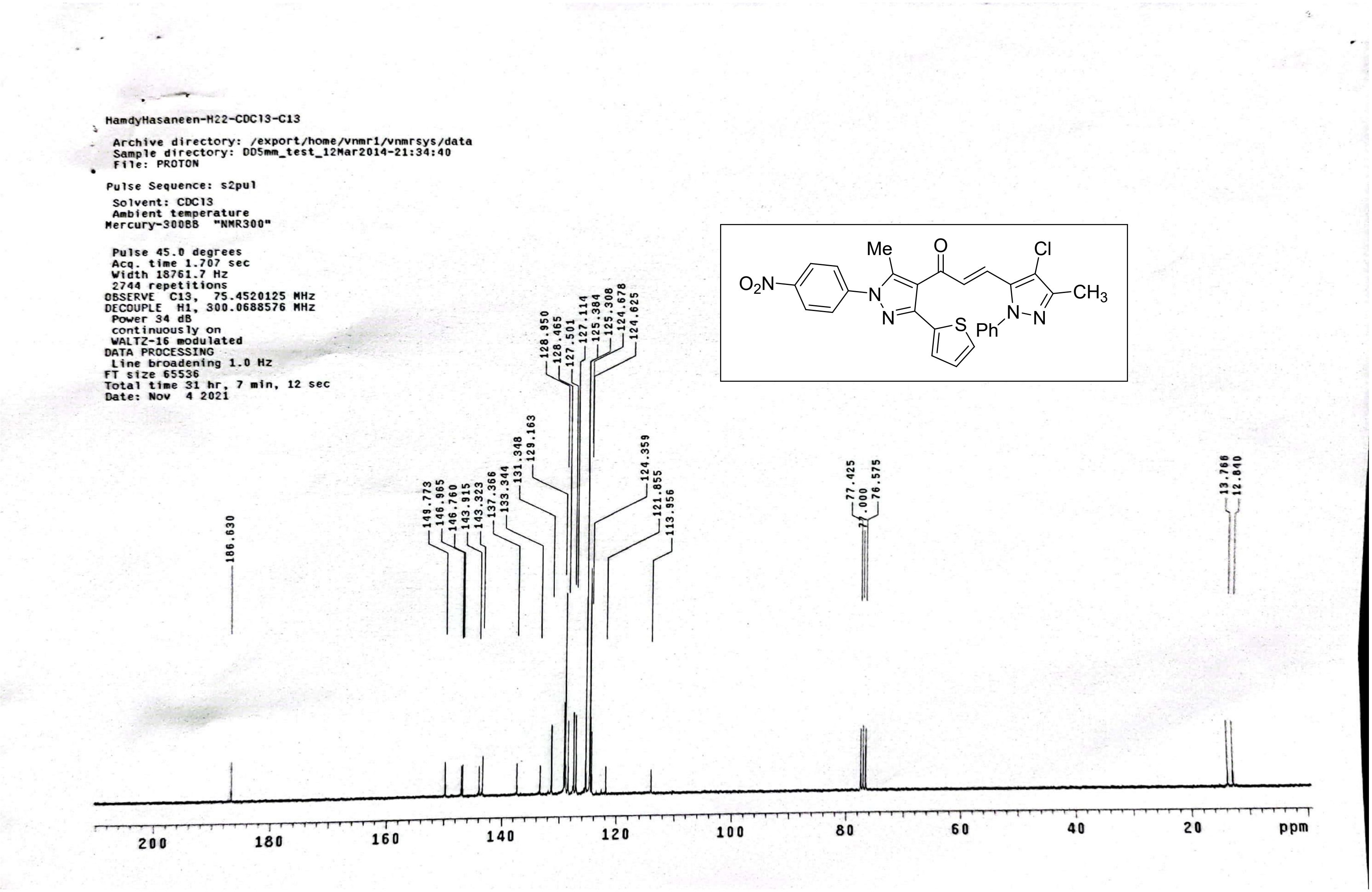

^{13}C-NMR spectrum of **7d**

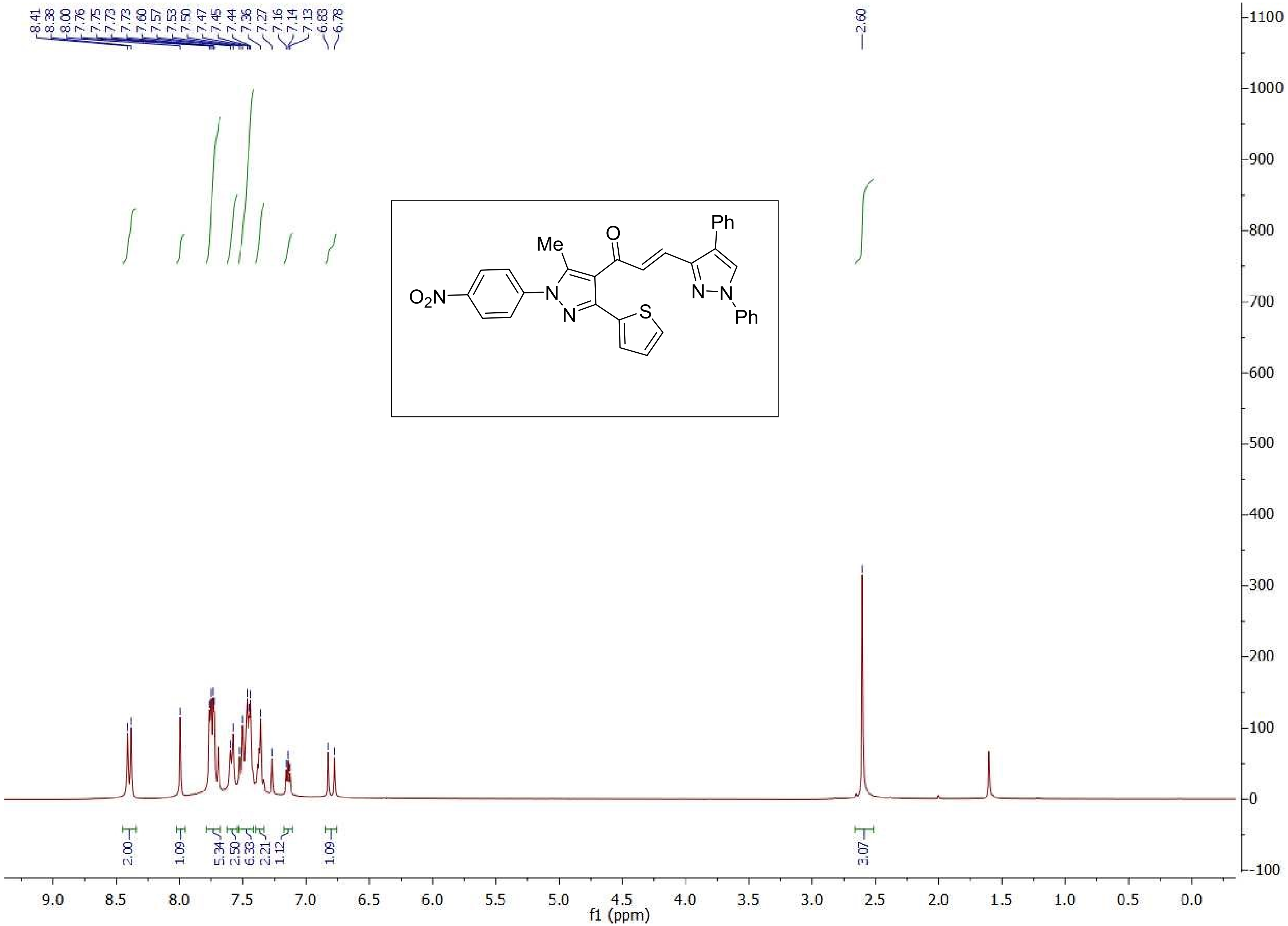

^{1}H-NMR spectrum of **9a**

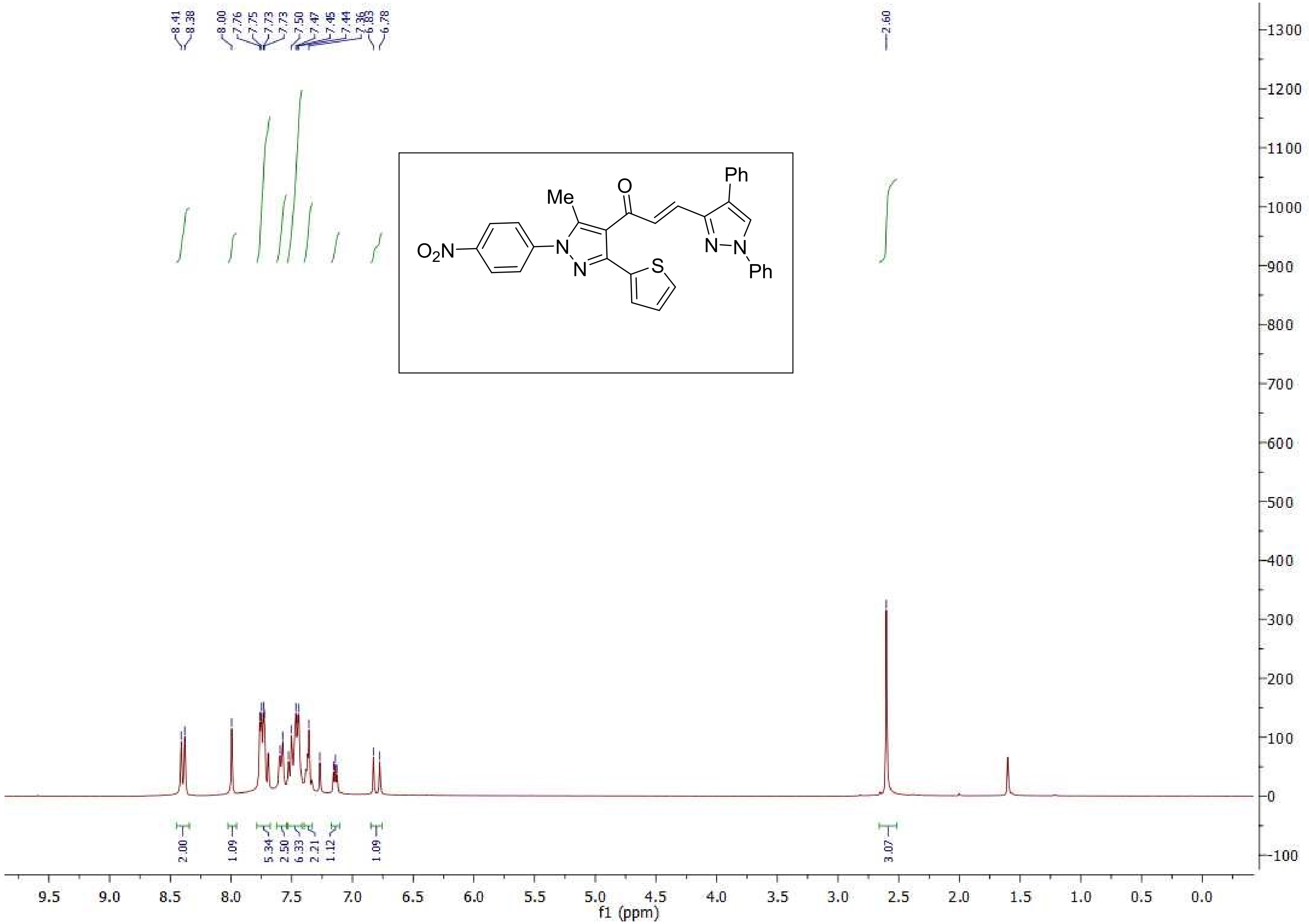

^{13}C-NMR spectrum of **9a**

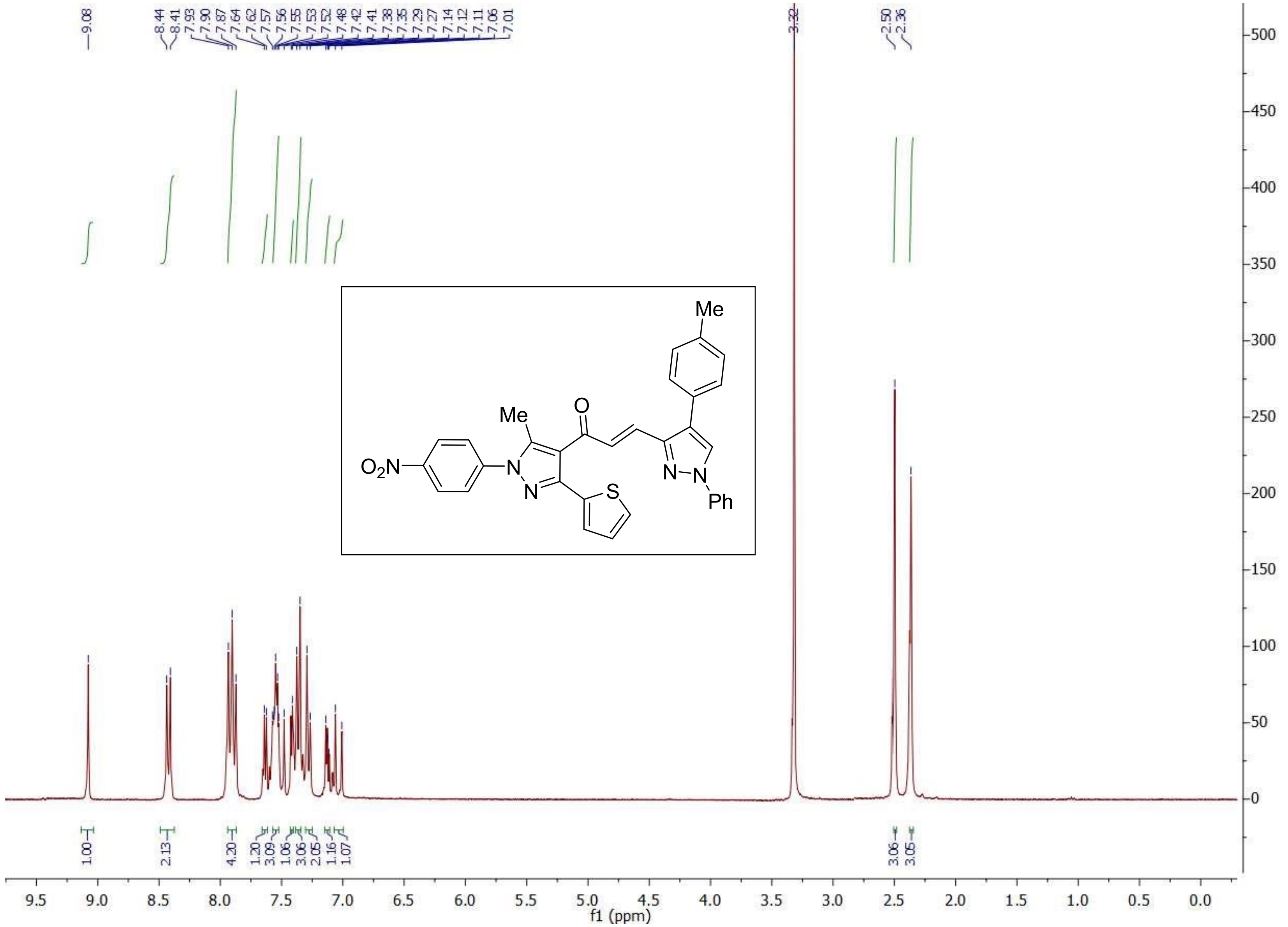

^{1}H-NMR spectrum of **9b**

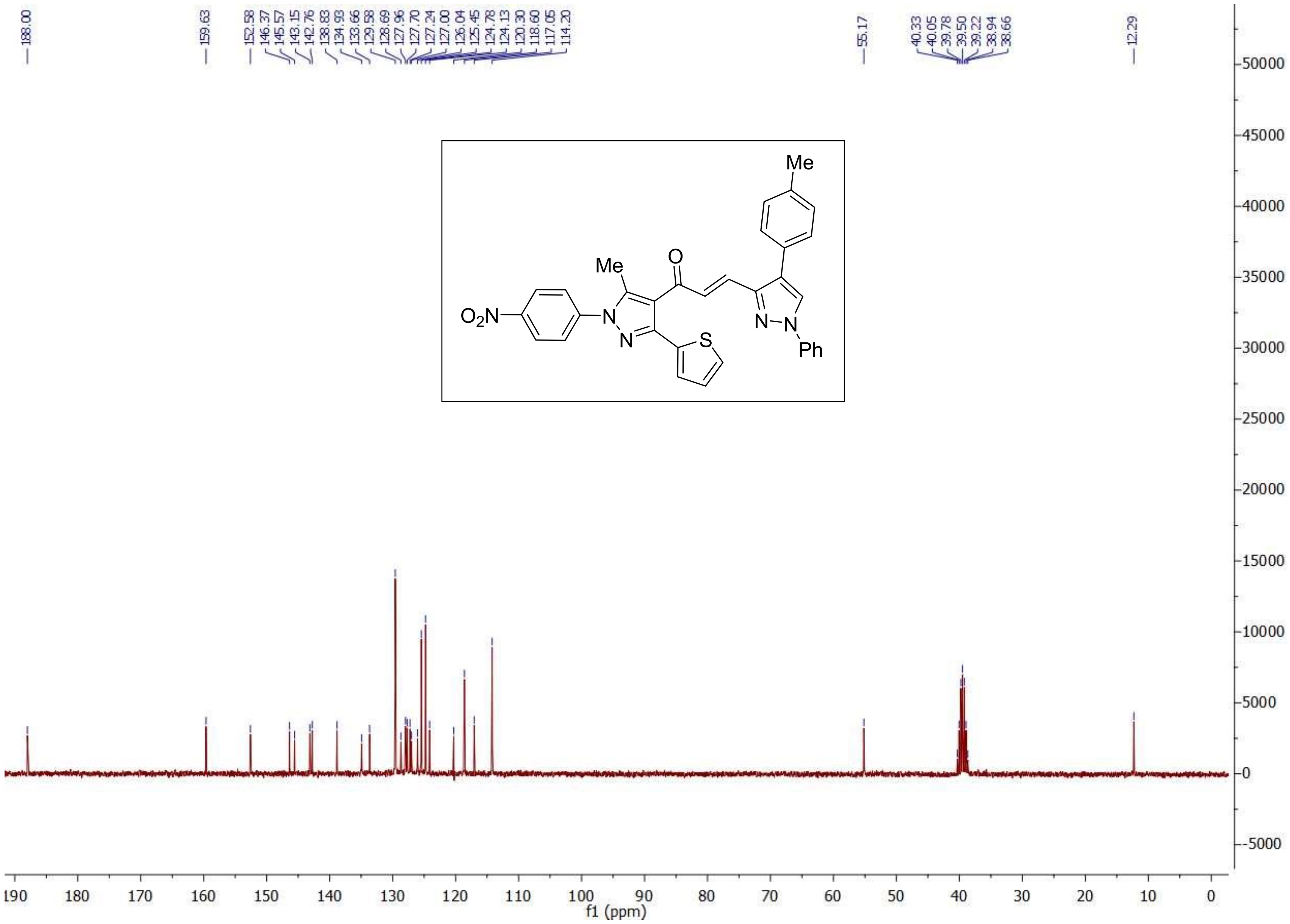

^{13}C-NMR spectrum of **9b**

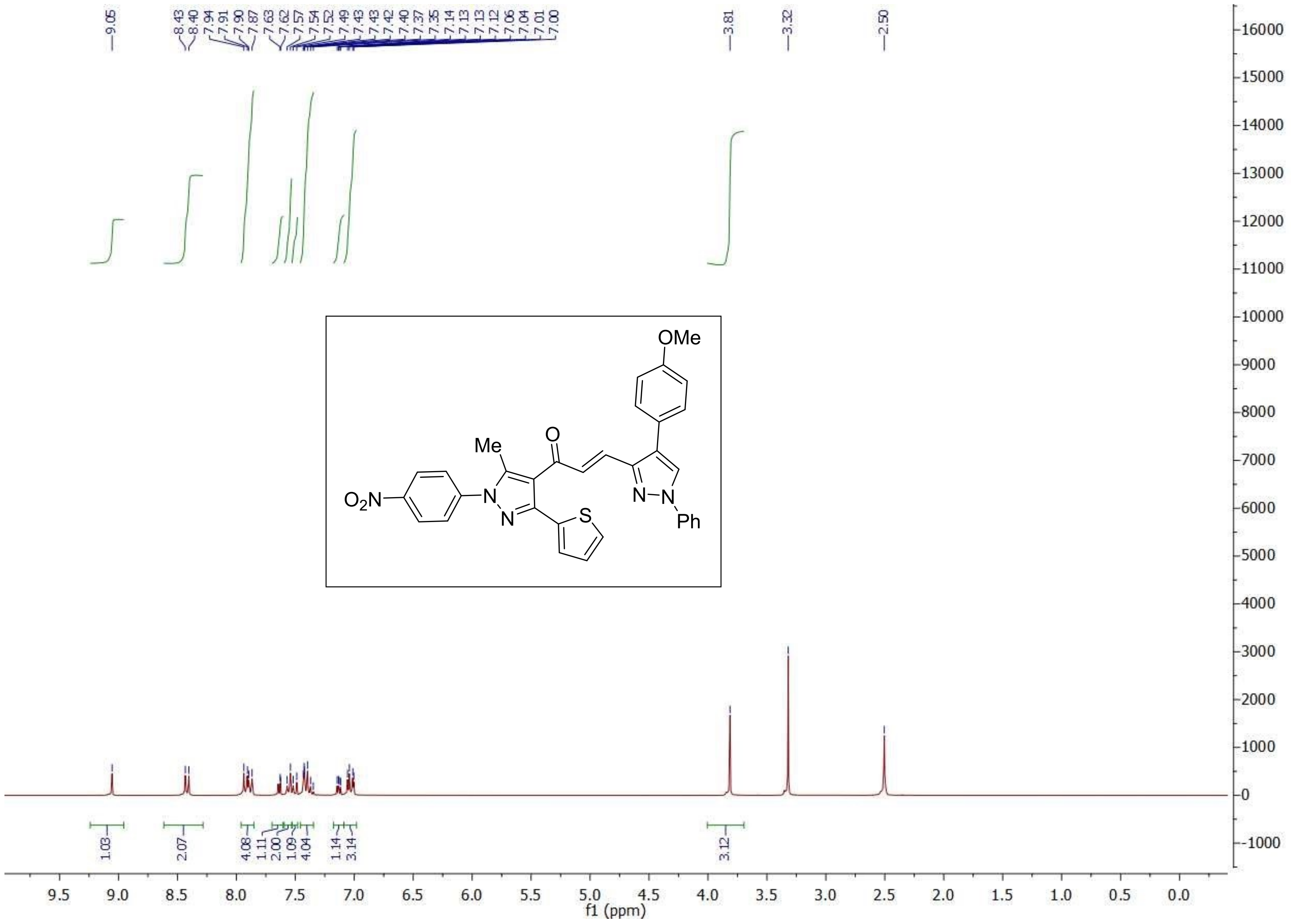

^{1}H-NMR spectrum of **9c**

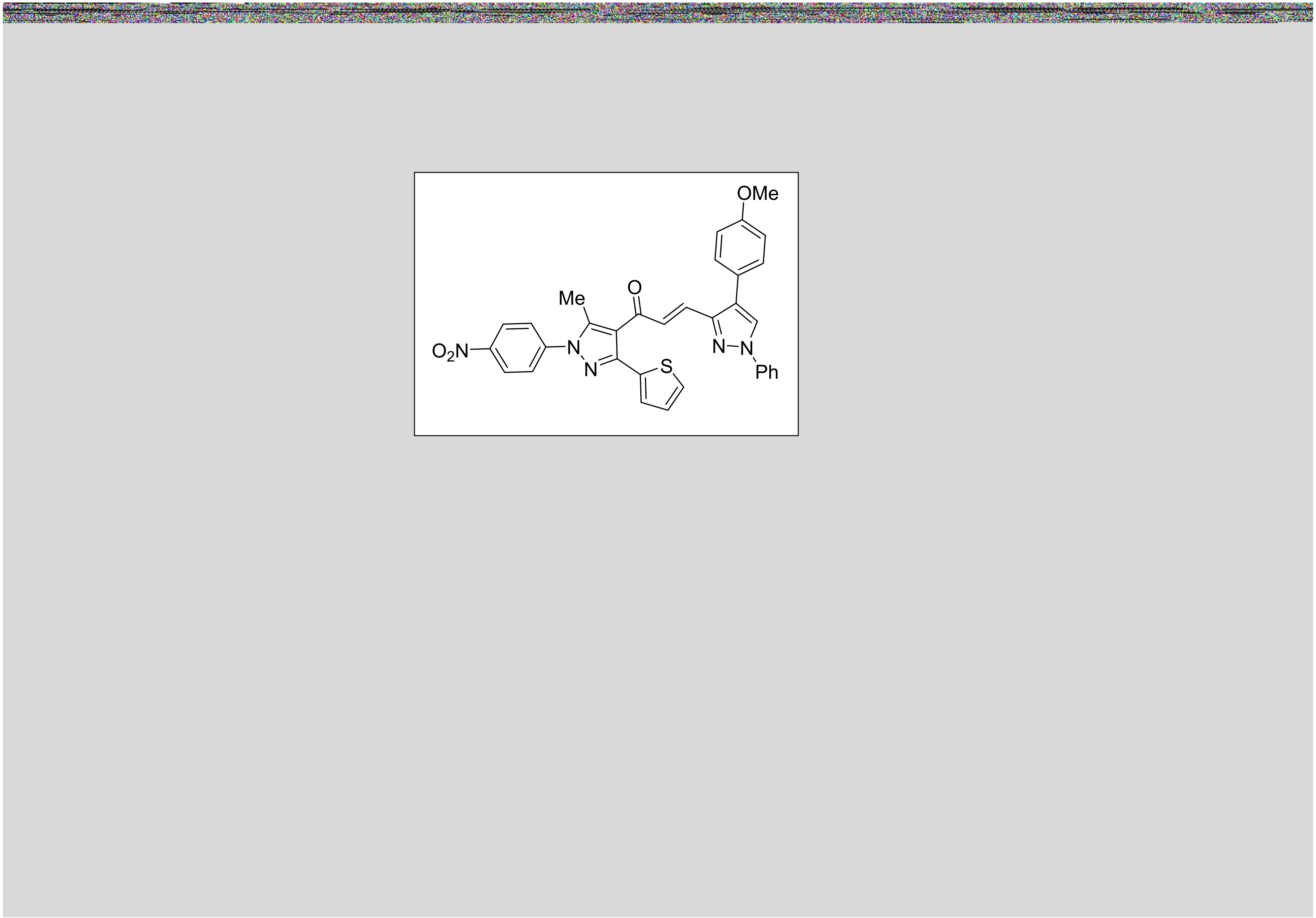

^{13}C-NMR spectrum of **9c**

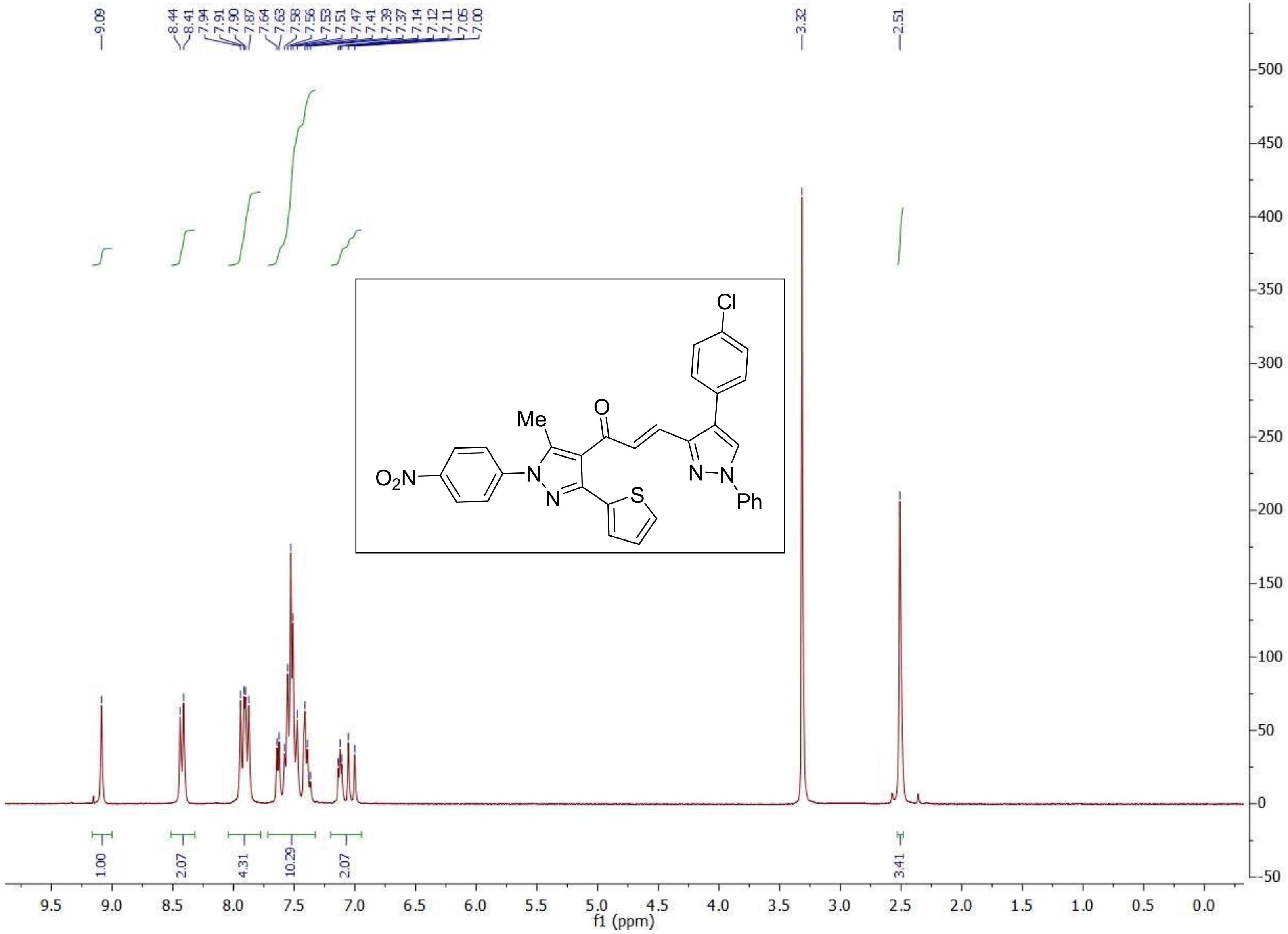

^{1}H-NMR spectrum of **9d**

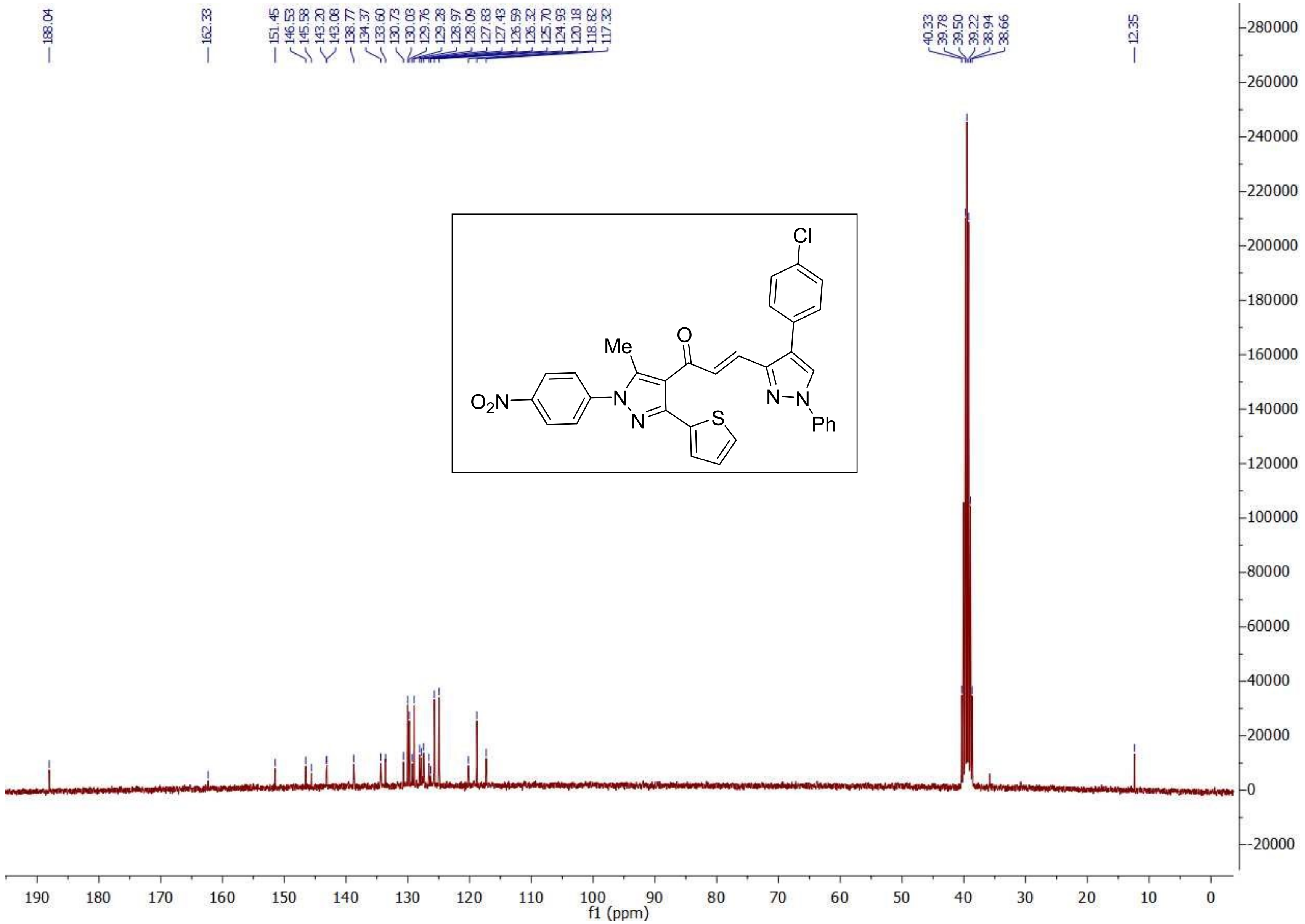

^{13}C-NMR spectrum of **9d**

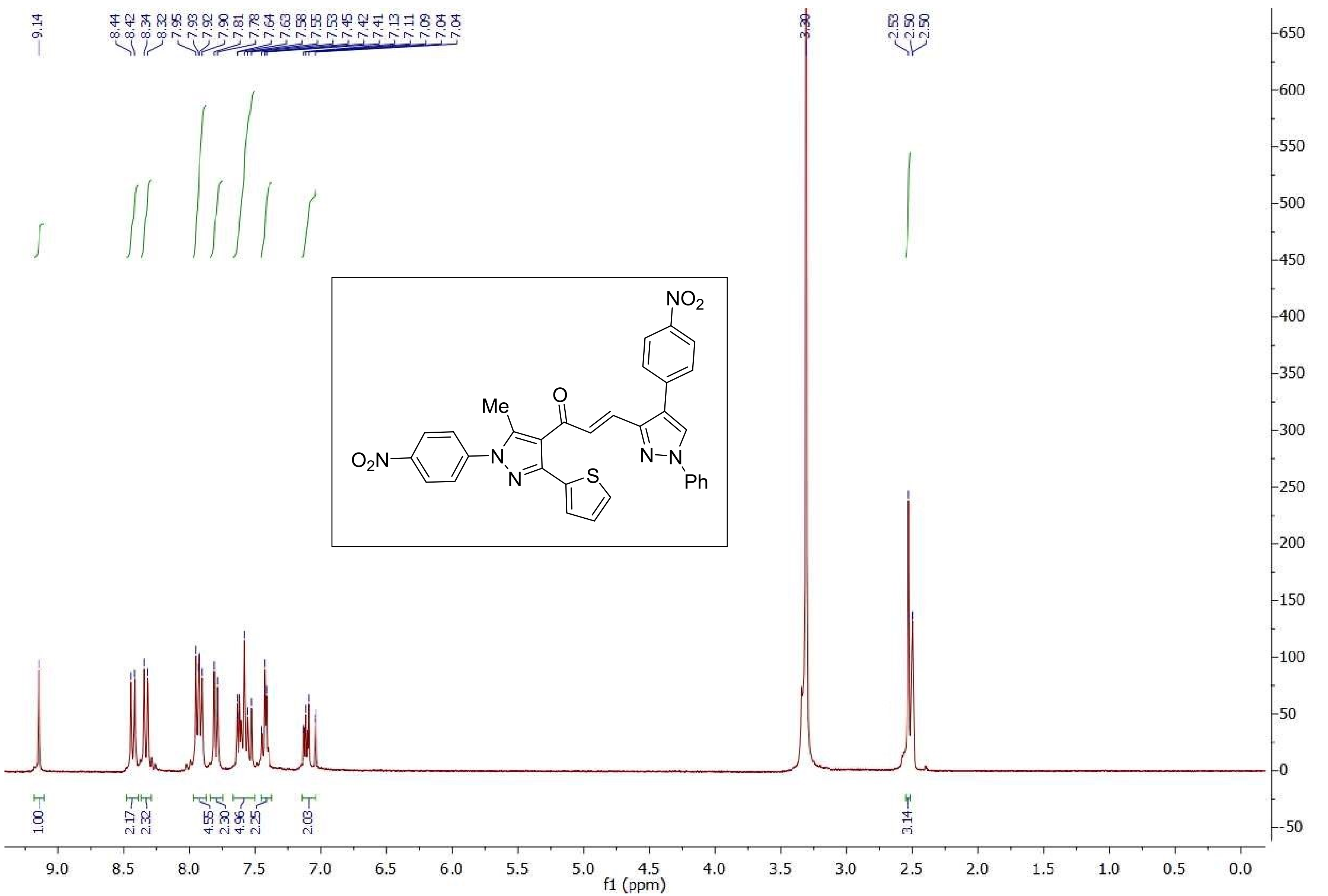

^{1}H-NMR spectrum of **9e**

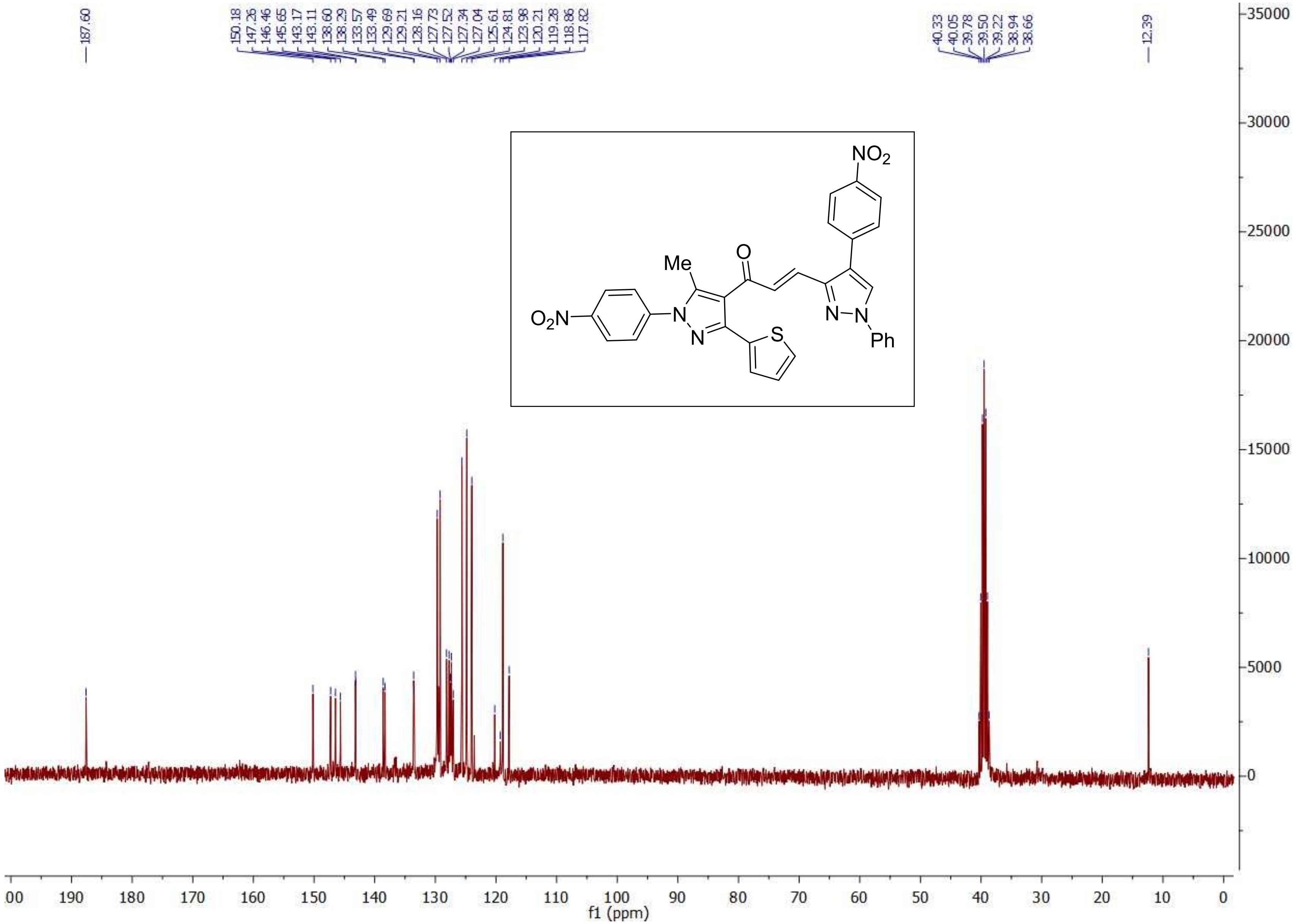

^{13}C-NMR spectrum of **9e**

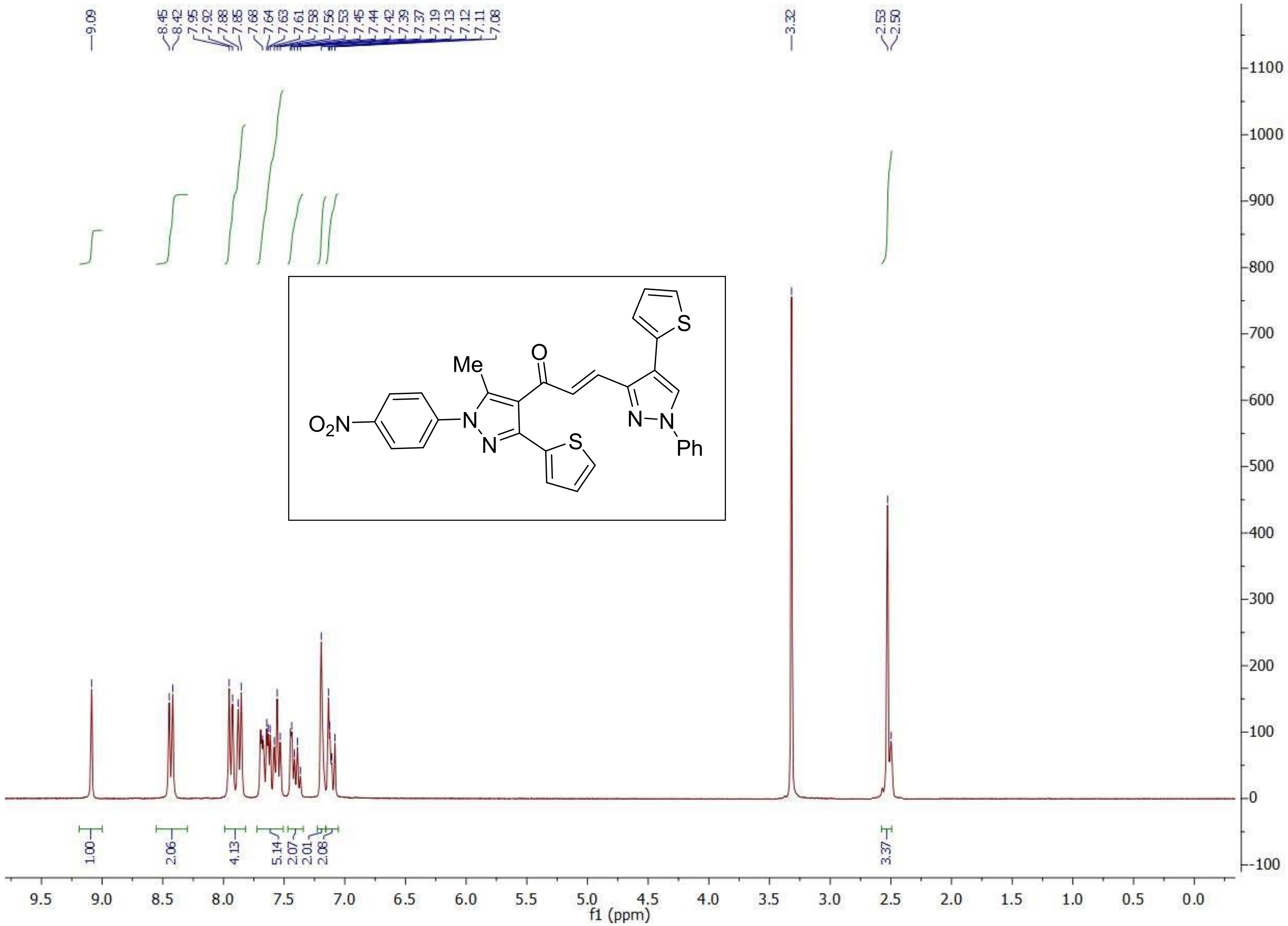

^{1}H-NMR spectrum of **9f**

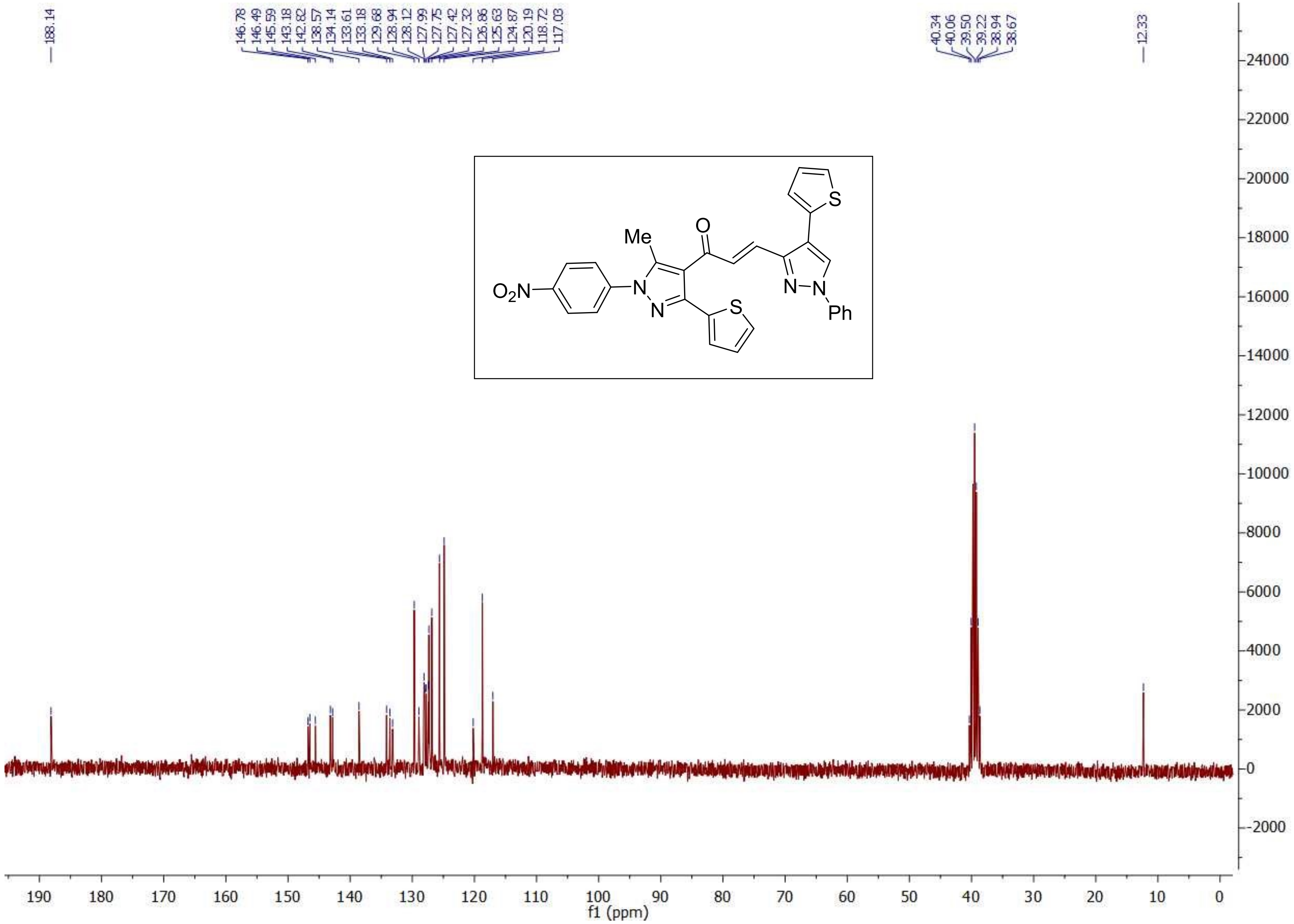

^{13}C-NMR spectrum of **9f**

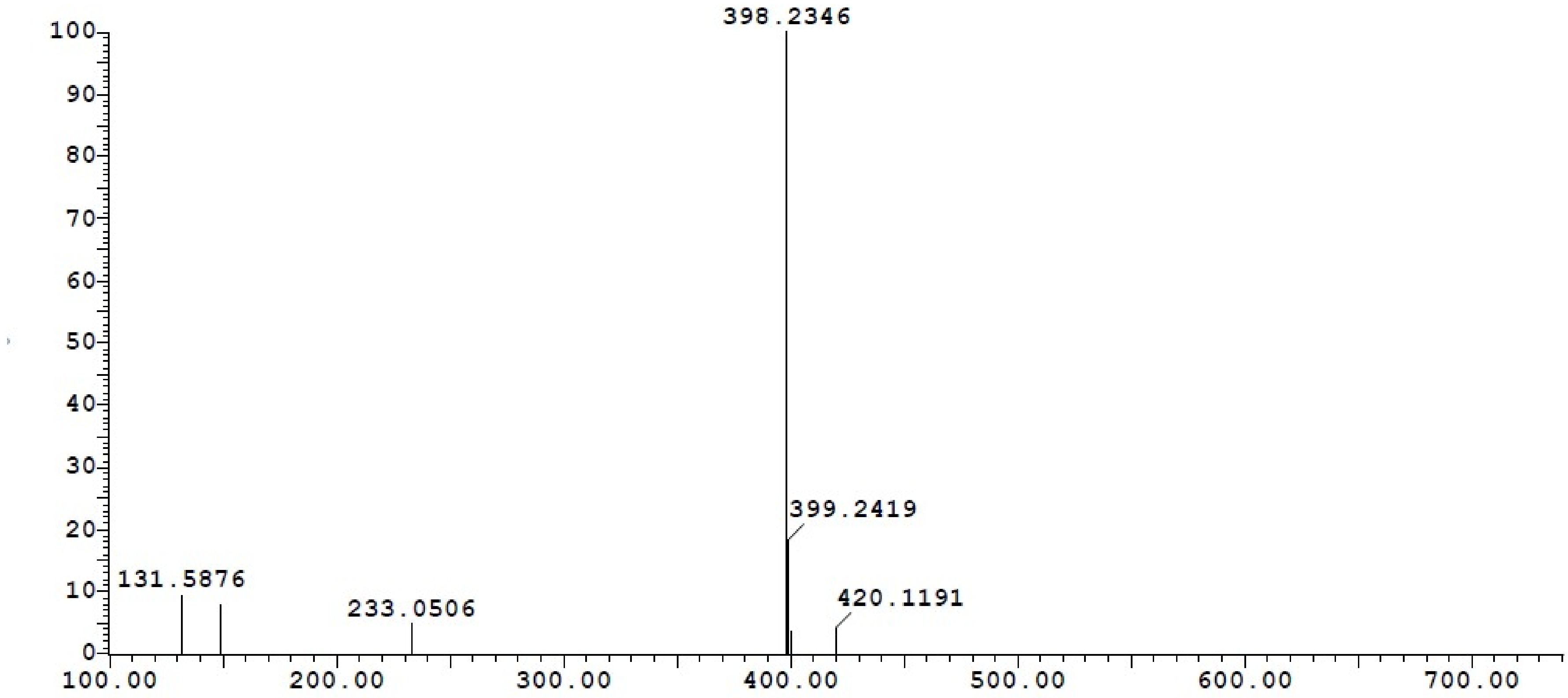

Mass spectrometry of **7b**

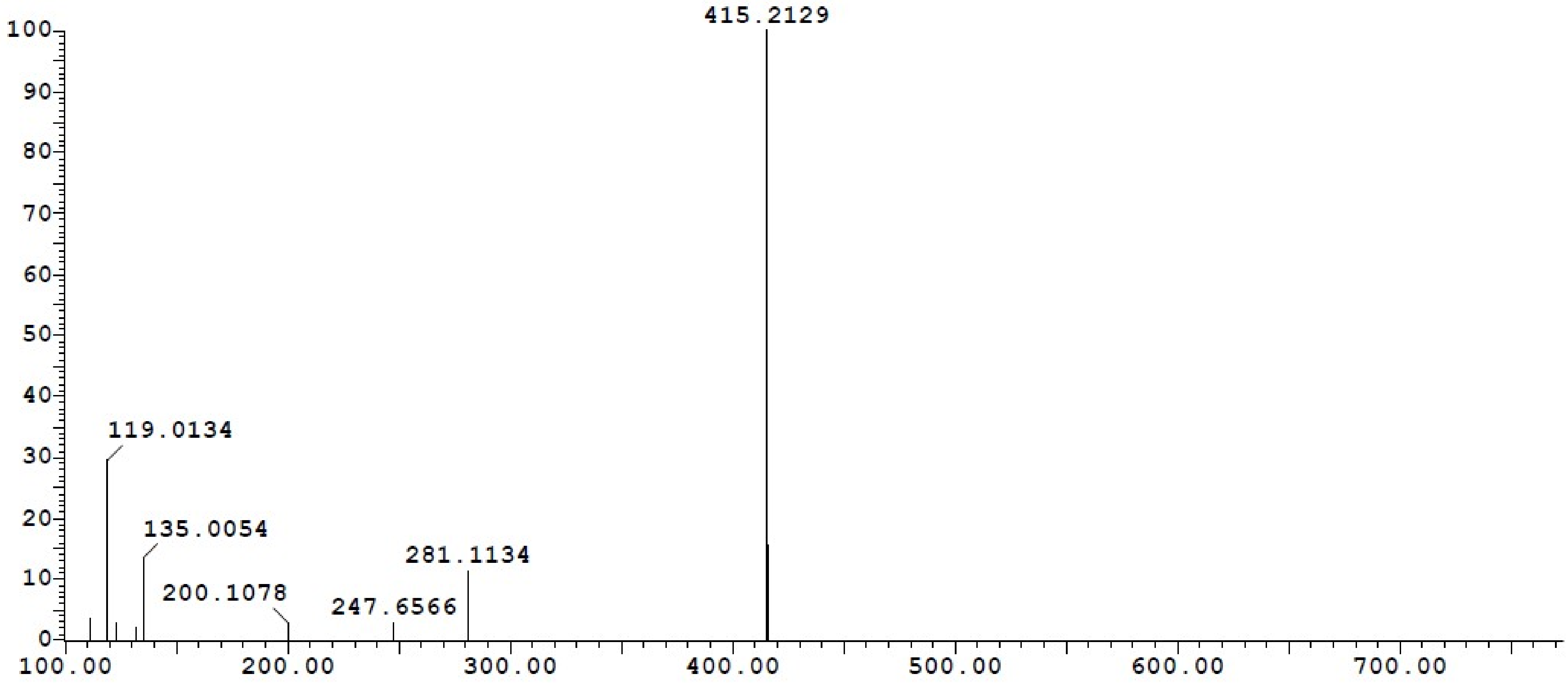

Mass spectrometry of **7c**

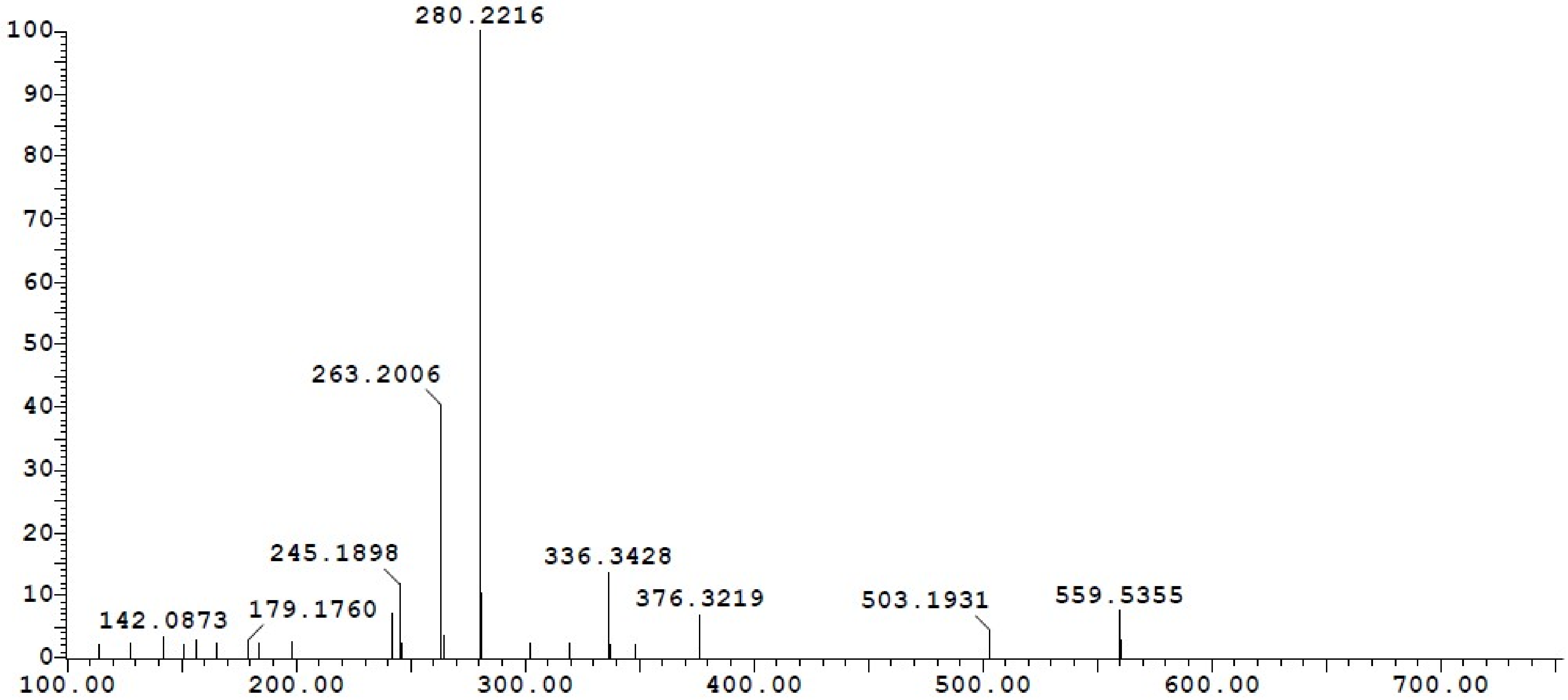

Mass spectrometry of **9a**

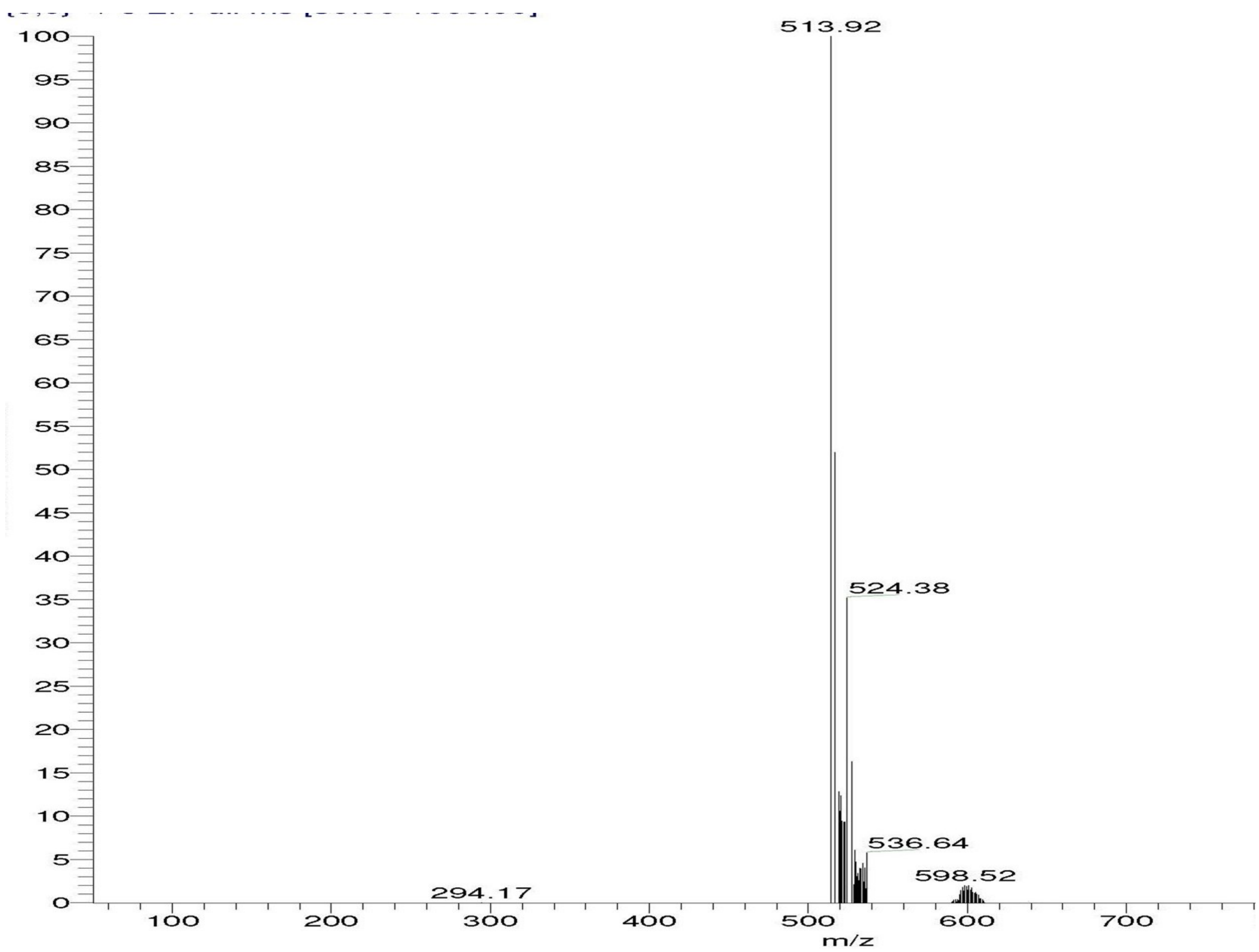

Mass spectrometry of **9d**

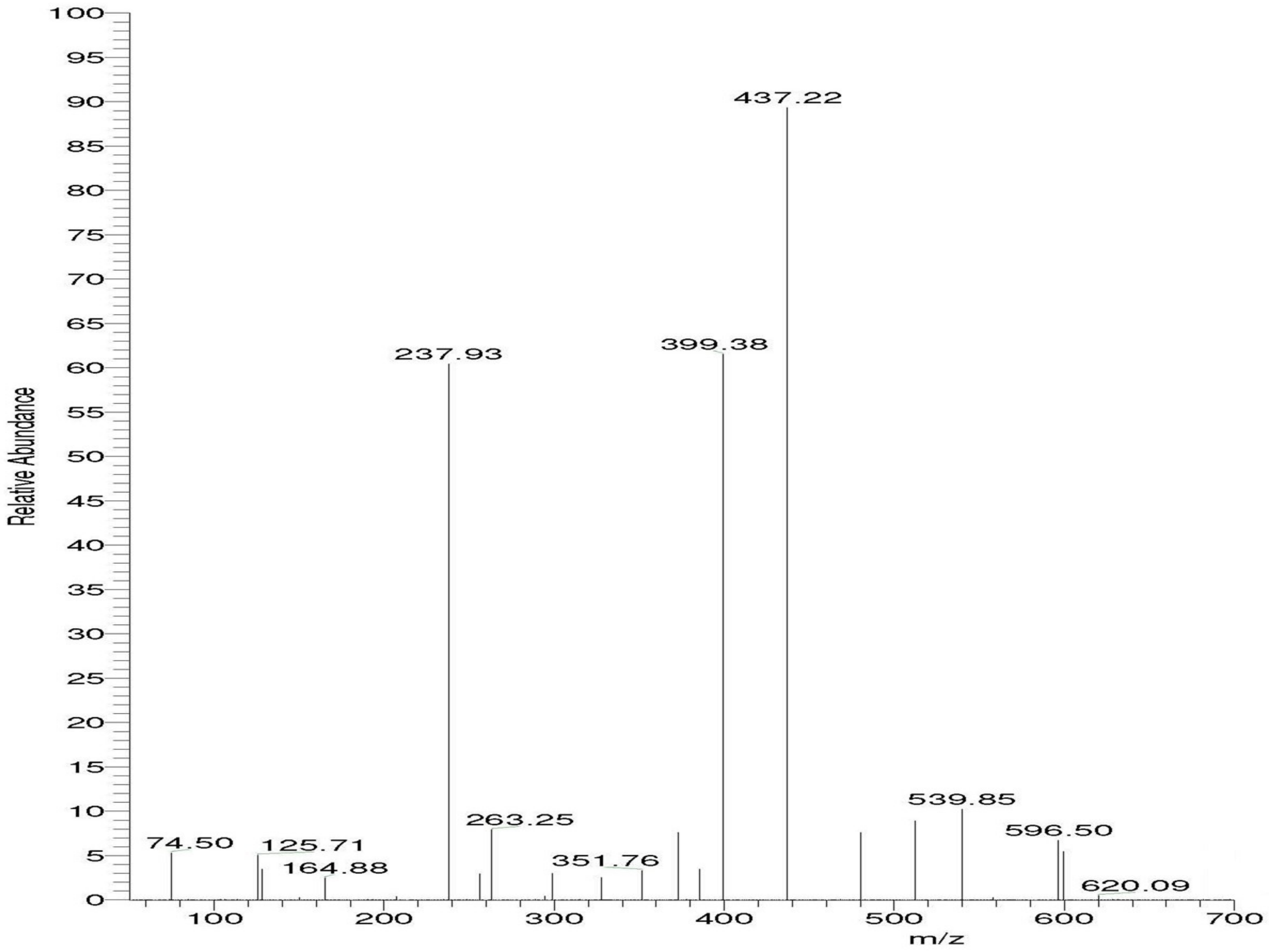

Mass spectrometry of **9e**

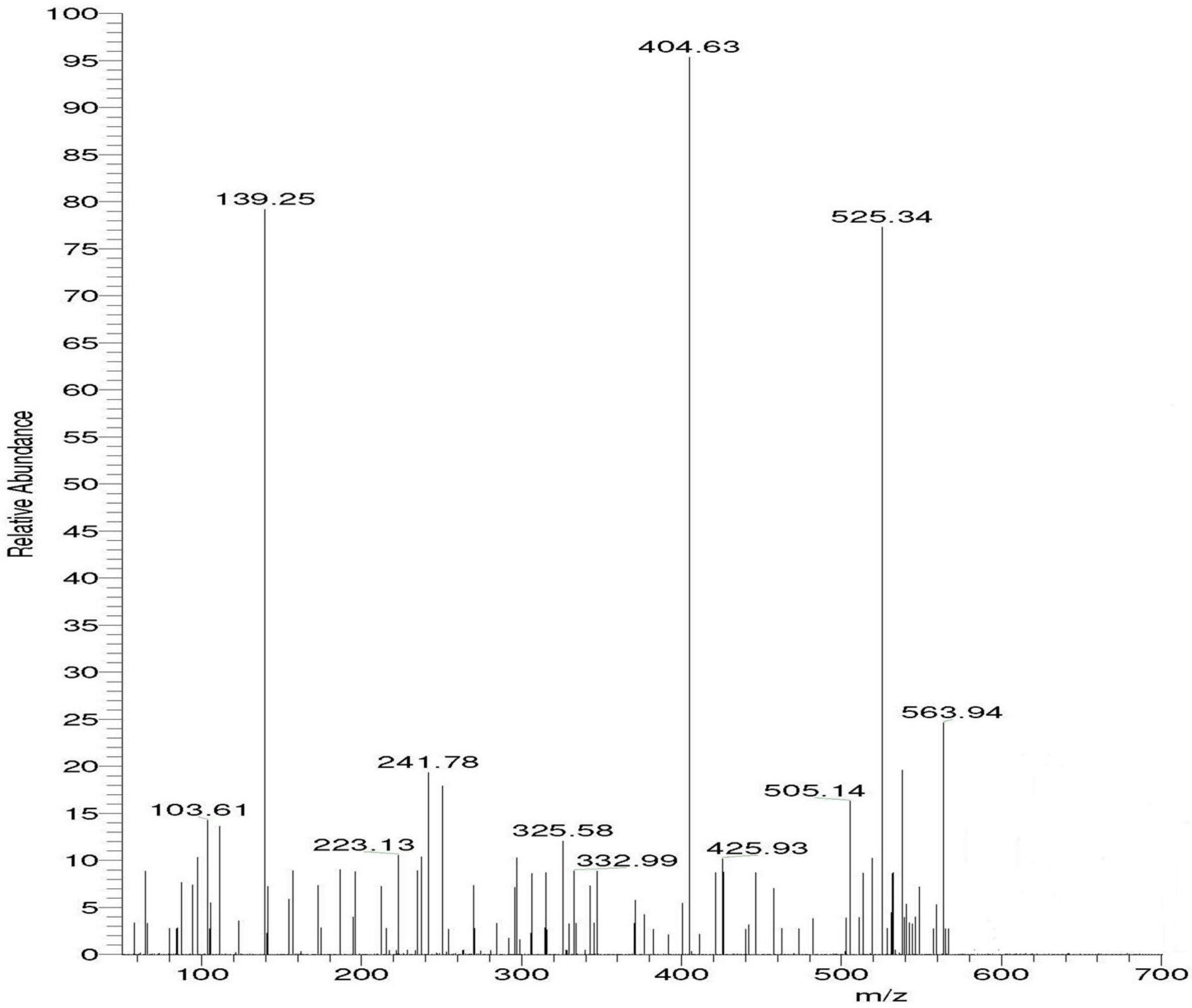

Mass spectrometry of **9f**
